# The Structure–Antimicrobial Activity Relationships of a Promising Class of the Compounds Containing the *N*-Arylpiperazine Scaffold †

**DOI:** 10.3390/molecules21101274

**Published:** 2016-09-26

**Authors:** Ivan Malík, Jozef Csöllei, Josef Jampílek, Lukáš Stanzel, Iveta Zadražilová, Jan Hošek, Šárka Pospíšilová, Alois Čížek, Aidan Coffey, Jim O’Mahony

**Affiliations:** 1 Department of Pharmaceutical Chemistry, Faculty of Pharmacy, Comenius University in Bratislava, Odbojárov 10, Bratislava SK-832 32, Slovak Republic; josef.jampilek@gmail.com (J.J.); lukasstanzel@gmail.com (L.S.); 2 Department of Chemical Drugs, Faculty of Pharmacy, University of Veterinary and Pharmaceutical Sciences in Brno, Palackého 1946/1, Brno CZ-612 42, Czech Republic; csolleij@cfu.cz (J.C.); 3 Department of Infectious Diseases and Microbiology, Faculty of Veterinary Medicine, University of Veterinary and Pharmaceutical Sciences in Brno, Palackého 1946/1, Brno CZ-612 42, Czech Republic; zadrazilova.iveta@seznam.cz (I.Z.); sharka.pospisilova@gmail.com (Š.P.); cizeka@vfu.cz (A.Č.); 4 Department of Molecular Biology and Pharmaceutical Biotechnology, Faculty of Pharmacy, University of Veterinary and Pharmaceutical Sciences in Brno, Palackého 1946/1, Brno CZ-612 42, Czech Republic; hhosek@gmail.com (J.H.); 5 Department of Biological Sciences, Cork Institute of Technology, Bishopstown, Cork T12 P928, Ireland; aidan.coffey@cit.ie (A.C.); jim.omahony@cit.ie (J.O.M.)

**Keywords:** *N*-arylpiperazines, *Mycobacterium kansasii*, *Mycobacterium marinum*, electronic properties, lipophilicity, structure–activity

## Abstract

This research was focused on *in silico* characterization and *in vitro* biological testing of the series of the compounds carrying a *N*-arylpiperazine moiety. The *in silico* investigation was based on the prediction of electronic, steric and lipohydrophilic features. The molecules were screened against *Mycobacterium avium* subsp. *paratuberculosis* CIT03, *M. smegmatis* ATCC 700084, *M. kansasii* DSM 44162, *M. marinum* CAMP 5644, *Staphylococcus aureus* ATCC 29213, methicillin-resistant *S. aureus* 63718, *Escherichia coli* ATCC 25922, *Enterococcus faecalis* ATCC 29212, *Candida albicans* CCM 8261, *C. parapsilosis* CCM 8260 and *C. krusei* CCM 8271, respectively, by standardized microdilution methods. The eventual antiproliferative (cytotoxic) impact of those compounds was examined on a human monocytic leukemia THP-1 cell line, as a part of the biological study. Promising potential against *M. kansasii* was found for 1-[3-(3-ethoxyphenylcarbamoyl)oxy-2-hydroxypropyl]-4-(3-trifluoromethylphenyl)piperazin-1-ium chloride (*MIC* = 31.75 μM), which was comparable to the activity of isoniazid (INH; *MIC* = 29.17 μM). Moreover, 1-{2-hydroxy-3-(3-methoxyphenylcarbamoyl)oxy)propyl}-4-(4-fluorophenyl)piperazin-1-ium chloride was even more effective (*MIC* = 17.62 μM) against given *mycobacterium*. Among the tested *N*-arylpiperazines, 1-{2-hydroxy-3-(4-methoxyphenylcarbamoyl)oxy)propyl}-4-(3-trifluoromethylphenyl)piperazin-1-ium chloride was the most efficient against *M. marinum* (*MIC* = 65.32 μM). One of the common features of all investigated substances was their insignificant antiproliferative (i.e., non-cytotoxic) effect. The study discussed structure–antimicrobial activity relationships considering electronic, steric and lipophilic properties.

## 1. Introduction

In medicinal chemistry, *N*-arylpiperazines have been regarded as so-called privileged substructures (PSs), i.e., they represent a class of the molecules capable of binding to multiple receptors or effector sites with a high affinity [1]. A systematic exploration of these compounds could allow promptly discover the biologically active ones across a broad range of therapeutic areas in a reasonable time scale. The PSs display very essential geometric, stereochemical, steric (including the size of the PS relative to the overall molecule) or physicochemical characteristics that facilitate their ability to bind to multiple receptors [2]. The *N*-aryl-/*N*-alkylpiperazine framework, as a key pharmacophore, attached to a 2-hydroxypropane-1,3-diyl connecting chain, which was bonded to a variously branched or a substituted lipophilic fragments directly or through a polar group, formed the structure of the compounds, which have shown *inter alia* promising potential to act against various *Mycobacterium* spp. [3,4,5], *Candida* spp. [6,7,8,9], *Staphyloccocus* spp. [10] or *Plasmodium* spp. [11] or might be the leads for further optimization and development [12,13].

**Figure 1:**
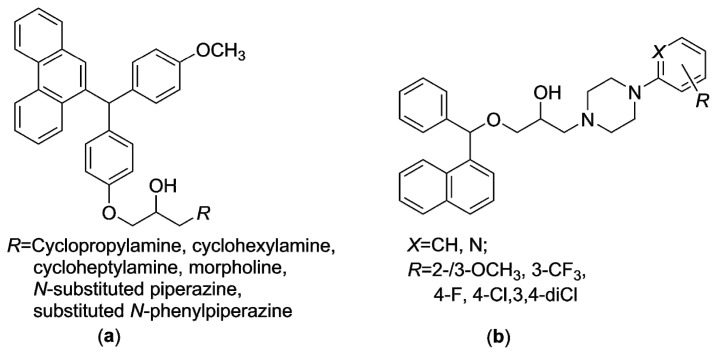
Highly lipophilic *N*-arylpiperazines, which contained: (**a**) a diaryloxymethanophenanthrene moiety; (**b**) a naphthalene moiety, have been promising compounds *in vitro* against *M. tuberculosis* H_37_R_v_ strain.

Regarding the structure–antimicrobial activity relationships (*SAR*) of some prospective derivatives, which structurally belong to that subclass of the *N*-arylpiperazines, it was revealed that the introduction of a 2-hydroxyaminoalkyl moiety on a lipophilic diaryloxymethanophenanthrene pharmacophore gave promising molecules, which have shown the *in vitro* activity against the *M. tuberculosis* H_37_R_v_ strain. In addition, an increase in a ring size of the amines led to effective compounds as well [3], as can be seen in Figure 1a. Steric factors apparently play an important role in antimycobacterial activity *in vitro* of the substances examined in the research [4]. As the steric bulk of the amines attached to “a core scaffold” decreased, the potency increased. Furthermore, the compounds carrying an electron-withdrawing substituent in the position 3 (3-CF_3_ or 3-NO_2_, for example), have shown very high antimycobacterial potential. On the contrary, the presence of an electron-donating OCH_3_ group in the 2-position led to the abolishment of the activity. When the group was moved to the 4-position, the effectiveness was found to be lower [4]. The introduction of a heterocyclic 4-(pyridin-2-yl)piperazin-1-yl moiety into the structure of the naphthalene derivatives based on mefloquine [5], as drawn in Figure 1b, meant the loss of antimycobacterial efficiency. Surprisingly, the *R* substituent with electron-donating properties (*R* = 2-/3-OCH_3_, *X* = CH) was considered favorable structural alternative, which contributed to an improvement in that activity [5]. In addition, a crucial hydrogen bonding interaction of a OH group with amino acid fragments at a binding site of a ATP synthase of *M. tuberculosis* H_37_R_v_ was observed *in silico* [5].

**Figure 2:**
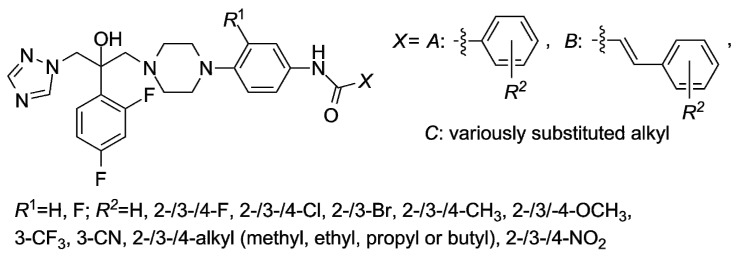
The *N*-arylpiperazine derivatives tested *in vitro* as the inhibitors of a lanosterol 14α-demethylase (CYP51).

Candidacidal properties *in vitro* of the compounds containing the 1*H*-1,2,4-triazol-1-yl and 4-(substituted phenyl)piperazin-1-yl moieties were also strongly influenced by the electronic, steric and lipophilic nature of the substituents attached to the piperazin-1,4-diyl fragment [6,7,8,9]. The proper selection of those *R*^1^, *X* and *R*^2^ substituents (Figure 2) was very essential for an interaction with appropriate effector sites of fungi (yeasts). Specifically, coordination bonds with iron of a heme group, hydrophobic and van der Waals interactions with complementary hydrophobic residues or π–π face-to-edge interactions were strongly taken into consideration [9].

The 4-*R*^2^- and 3-*R*^2^-substituted derivatives have shown higher antifungal activity compared to the 2-*R*^2^-substituted compounds (*X* = *A* or *B*). A docking model indicated that the active site of a lanosterol 14*α*-demethylase from *Candida albicans* (CACYP51) at the position 2 of a specific bound compound was not large enough to accommodate an additional group [6,8]. The antifungal potency was decreased when the lipophilicity was increased “above a certain” value, i.e., if the *X* substituent (Figure 2) was butyl or higher alkyl group [9].

Besides, the antimicrobial potential of previously studied *N*-aryl-/*N*-arylalkylpiperazines or their synergistic effect with other antimicrobials [14,15,16,17,18] could be considered very promising when taking into account the development and spreading of antibiotic resistance in bacteria or a resistance in fungi (yeasts) as a universal severe threat to both humans and animals.

In the first part of this study, which preceded another presented phase, an *in vitro* biological evaluation of lipophilic *N*-arylpiperazine derivatives **5a**–**l** (chemically 1-[3-(2-/3-/4-alkoxyphenyl-carbamoyl)oxy-2-hydroxypropyl]-4-(3-trifluoromethyl-/4-fluorophenyl)piperazin-1-ium chlorides), an attention was paid on their *in silico* characterization to closely investigate their electronic, steric and lipophilic properties. Supposed differences in those features would result from the presence (combination) of both substituents attached to their lipophilic and salt-forming parts (Table 1) and might influence the compounds′ biological activity.

In addition, compared to the molecules drawn in Figure 1, the 2-/3-/4-alkoxyphenylcarbamoyloxy fragment was chosen as “a more hydrophilic” alternative to a highly lipophilic acyloxy moiety consisting of five (Figure 1a), or three (Figure 1b) aromatic systems. Given modification led to the compounds **5a**–**l**, which probably were not be able to cross a blood-brain barrier by a passive process and involve CNS side effects [19].

Atomic/fragmental and whole-molecule calculation procedures were employed in order to describe compounds’ lipohydrophilic properties in a more detailed way and find acceptable as well as less time-consuming *in silico* alternatives to an experimental evaluation.

Next, a very essential objective of current research was the *in vitro* screening of those molecules against various strains of the mycobacteria, Gram-positive and Gram-negative bacterial strains and the yeasts, respectively, with an ambition to find some promising antimicrobially effective substances. Furthermore, an eventual antiproliferative (cytotoxic) effect of all the derivatives was *in vitro* tested against a human monocytic leukemia THP-1 cell line to preliminary explore a possible relation between the capability to inhibit proliferation (i.e., between the cytotoxicity) and the antimicrobial activity.

After the *in silico* examination and the *in vitro* biological testing, a key aim of the research was to reveal some structural and physicochemical features of the compounds **5a**–**l**, which might appear to be essential for their antimicrobial efficiency and thereby to contribute to comprehensive structure–antimicrobial activity relationships analyses in this class of the compounds.

## 2. Results

### 2.1. Electronic, Steric and Lipohydrophilic Properties of the Compounds ***5a**–**l***

The *N*-arylpiperazine compounds under current study were “virtually” divided into two main classes according to the electronic, steric and lipohydrophilic features of the substituents attached to the 4′-(3′-/4′-substituted phenyl)piperazin-1′-yl moiety (Table 1). Fluorine-containing group(s) play a pivotal role in a drug discovery process for modulating the antimicrobial efficiency of molecules. The effects of a fluorine substitution in organic compounds include the ability of that atom to participate in a hydrogen bonding, either as a hydrogen-bond acceptor or as an inductive activator of a hydrogen-bond donor group. The fluorine substitution on aromatic substructures renders the remaining aromatic hydrogen substituents more acidic, so the capacity of those compounds to act as hydrogen bridge donors is enhanced [4,5,6,7,8,9,20,21,22]. In addition, non-covalent intermolecular interactions of the C–F bond can be important for the affinity of a drug with a macromolecular recognition site [20].

The Group I compounds included the derivatives **5a**–**f** carrying the 3′-CF_3_ group, which has shown strong electron-withdrawing effect. The electronic properties might also be described by the Hammett substituent constant *σ*; a more positive value of *σ* means a stronger electron-withdrawing influence of the substituent [23]. The *σ* output for the 3′-CF_3_ moiety (*σ*_CF3_) was set to 0.43, the value for hydrogen was *σ*_H_ = 0.00. The Group II molecules **5g**–**l** contained the 4′-F atom, which has been known to be slightly electron-withdrawing, as the readout *σ*_F_ = 0.06 indicated [23]. Electronic properties of all the studied *N*-arylpiperazines could be also characterized by the values of their dissociation constants [23]. For the series **5a**–**l**, the p*K*_a_s were estimated by a potentiometric titration [24,25] and were listed in Table 1.

It was found out that the presence of the 3′-CF_3_ group provided lower values of the compounds′ p*K*_a_, which were observed in the range from 5.35 (the compound **5d**) to 6.00 (**5b**). The positional isomers **5g**–**l** carrying the 4′-F substituent have shown moderately higher p*K*_a_s in the interval from 6.31 (**5j**) to 7.24 (**5k**). The elongation of an alkoxy side chain led to lower p*K*_a_s of the Group II derivatives; that course was not observed among the Group I molecules. The highest p*K*_a_ readouts were estimated for the 4-alkoxy substituted compounds **5k** (p*K*_a_ = 7.24) and **5l** (7.18), respectively (Table 1).

The values of representative steric descriptors *L*, *B*_i_–*B*_iv_ [23] proved that the 3′-CF_3_ moiety was sterically almost twice as bulky than the 4′-F one. The differences in the steric properties of the inspected bases **5aB**–**lB** (Scheme S2 in Supplementary Materials) were verified by calculation of their molecular volume (*MV*) using an interactive Molecular Properties Calculator applet (MolSoft L.L.C. San Diego, CA, USA). In accordance with theory, the substances, which belonged to the Group I, were sterically bulkier than their positional isomers from the Group II. In addition, a decrease in the *MV*s was observed in both Groups I and II, as follows: 2-positional isomers > 3-positional isomers > 4‑positional isomers (Table 1). The highest *MV* value was related to the compound **5bB** (436.81 Å^3^), in which structure the *R*^1^ = 2-OC_2_H_5_ and *R*^2^ = 3′-CF_3_ substituents were attached to. On the other hand, the lowest *MV* was calculated for the molecule **5kB** (386.77 Å^3^), which contained the *R*^1^ = 4-OCH_3_ and *R*^2^ = 4´-F groups. The applet did not allow generate the data for the final salts **5a**–**l**, but it could be presumed that the suggested tendency would be maintained.

The lipophilicity *π* constant related to the CF_3_ group, which was directly attached to aromatic system, was 0.88 [23]. This value was higher than the readout for the F atom (*π* = 0.14). In addition, the position (i.e., the positional isomerism) of an alkoxy side chain influenced electronic and steric properties as well as the lipophilicity of the presently studied derivatives **5a**–**l**. That feature affected their *in vitro* activity, especially against *M. kansasii* DSM 44162 and *M. marinum* CAMP 5644. Those impacts were described in further sections of the paper.

The lipophilicity has been considered one of the essential factors involved in structure–anti-microbial activity relationship studies [26,27,28,29]. Experimentally observed values of the partition coefficient for the molecules **5a**–**l** in the octan-1-ol/phosphate buffer (p*H* = 7.4) system (log *P*_exp_s) indicated their highly lipophilic nature [24,25,30]. Those log *P*_exp_s ranged from 3.25 (**5i**) to 3.90 (**5h**; Table 2).

The current research provided the log *P* data calculated *in silico* by four atomic/fragmental methods, namely CLOGP 4.0 [31], Ghose and Crippen′s log *P*_Cf_ [32,33,34], Viswanadhan′s log *P*_Vf_ [35] and Broto’s log *P*_Bf_ [36]. In addition, the ALOGPs predictor [37] based on a whole-molecule approach was used as well. According to all the methods, the increase in lipophilicity of analyzed derivatives resulted in higher log *P*s (Table 2). Positional isomerism of an attached alkoxy side chain was not being reflected in the log *P* outputs, which were generated by the approaches based on a substructure principle. Following the readouts from all the predictive procedures, the Group I molecules were more lipophilic than their positional isomers, which were included in the Group II. The highest calculated level of the compounds′ lipophilicity was connected with CLOGP 4.0 and that values were in an interval from 3.34 to 4.74. On the other hand, the lowest log *P*s were generated by the log *P*_Bf_ method, which outputs varied from 1.67 to 2.86 (Table 2). Following those results, the derivatives **5g**, **5i** and **5k** were moderately lipophilic, with log *P*_Bf_ = 1.67.

Before inspecting the observed differences, it should be admitted that the rather small number of tested molecules confined a generalization of such validity comparisons. On the basis of the *F* values (Fisher’s *F*-test) calculated by the Origin Pro 9.0.0 software (OriginLab Corporation, Northampton, MA, USA), following ranking of those five included calculation programs was observed for the dataset of non-protonated bases **5aB**–**lB**: CLOGP 4.0 (*F* = 3.23) > log *P*_Vf_ (*F* = 2.94) > ALOGPs (*F* = 2.92) > log *P*_Cf_ (*F* = 2.91) > log *P*_Bf_ (*F* = 2.83). According to the averaged absolute residual sums *AARS* [38], following ranking of those programs was observed: log *P*_Vf_ > log *P*_Cf_ > CLOGP 4.0 > ALOGPs > log *P*_Bf_ (Table 3). The classification into acceptable, disputable and unacceptable calculations mirrored that view. Counting the negative and positive deviations of calculations from the log *P*_exp_s has shown a rather equilibrated pattern for both acceptable log *P*_Vf_ and log *P*_Cf_ (Table 3).

Only the ALOGPs procedure allowed to analyze protonated forms of investigated derivatives. In fact, when using the ALOGPs, one nitrogen atom of a piperazin-1,4-diyl fragment was protonated and, in addition, chlorides were not included in the calculations. However, statistical *F* data (0.02) as well as the *AARS* (1.02) indicated that the given possibility was completely unacceptable. Moreover, it was not possible to completely generate the outputs related to the majority of the reference drugs RIF, CPX, AMP or Amph. B (Table 2) due to some limitations, which resulted from the limits of the various algorithms implemented in chosen predictive approaches. Among the standards, the most lipophilic substance was RIF (CLOGP 4.0 = 4.03, ALOGPs = 2.35). On the other hand, INH, CPX, 5-FC, AMP as well as Amph. B were regarded as hydrophilic standard drugs with CLOGP 4.0 < −0.67 and ALOGPs < 0.88, respectively (Table 2).

### 2.2. Biological Assays in vitro

#### 2.2.1. Antimicrobial Susceptibility Testing

In the light of the current *in vitro* antimycobacterial screening (Table 4), the derivatives **5a**–**l** were weakly active against a clinical isolate of *M. avium* subsp. *paratuberculosis* CIT03 with the minimum inhibitory concentration (*MIC*) values ranging from 510.29 μM (substance **5a**) to 2273.19 μM (**5g** and **5k**). Similarly, the compounds were practically inactive against *M. smegmatis* ATCC 700084 with observed *MIC*s from 127.00 μM (**5d**) to 581.94 μM (**5g**, **5i** and **5k**). Only the most lipophilic derivative from the Group I, 1-[3-(3-ethoxyphenylcarbamoyl)oxy-2-hydroxypropyl]-4-(3-trifluoromethyl-phenyl)piperazin-1-ium chloride (**5d**; log *P*_exp_ = 3.72, log *P*_Cf_ = 3.95 and log *P*_Vf_ = 4.06), has shown a moderate effect against *M. smegmatis* (*MIC* = 127.00 μM) and which activity was comparable to the potential of INH (*MIC* = 117.03 μM). From a whole set of investigated molecules, the RIF standard was the most active (*MIC* = 19.40 μM).

The lowest *MIC*s were observed when inspecting the *in vitro* activity of the substances **5a**–**l** against *M. kansasii* DSM 44162 and *M. marinum* CAMP 5644. In general, the Group I derivatives were more efficient against both mycobacterial strains than their positional isomers from the Group II, excluding the molecules **5d** and **5j** (Table 4).

The compound 1-[3-(3-ethoxyphenylcarbamoyl)oxy-2-hydroxypropyl]-4-(4-fluorophenyl)-piperazin-1-ium chloride (**5j**) was the most prospective against *M. kansasii* (*MIC* = 17.62 μM) and it was even more effective than the reference drug INH (*MIC* = 29.17 μM). Comparable potential to INH to fight against mentioned *mycobacterium* was also found for 1-[3-(3-ethoxyphenylcarbamoyl)oxy-2-hydroxypropyl]-4-(3-trifluoromethylphenyl)piperazin-1-ium chloride (**5d**; *MIC* = 31.75 μM). Furthermore, the compound 1-{2-hydroxy-3-(4-methoxyphenylcarbamoyl)oxy)propyl}-4-(3-tri-fluoromethylphenyl)piperazin-1-ium chloride (**5e**) was slightly less efficient (*MIC* = 65.32 μM). The *N*-arylpiperazines, which have shown promising antimycobacterial activity, were highlighted in gray (Table 4). Regarding only the position of the *R*^1^ substituent, it was found that the 3-alkoxy derivatives were more active against *M. kansasii* than their 2- or 4-alkoxy positional isomers, excluding the substance **5e**.

The factor of a positional isomerism was also reflected in the action of the molecules **5a**–**l** against *M. marinum*. The compound **5e** was the most efficient (*MIC* = 65.32 μM), and its activity was higher than the potency of INH (*MIC* = 466.68 μM), but notably lower compared to the efficiency of RIF (*MIC* = 2.43 μM; Table 4).

A common denominator in the present *in vitro* screening of the compounds **5a**–**l** against *Staphylococcus aureus* ATCC 29213, methicillin-resistant *S. aureus* 63718, *Escherichia coli* ATCC 25922, *Enterococcus faecalis* ATCC 29212, *Candida albicans* CCM 8261, *C. parapsilosis* CCM 8260 and *C. crusei* CCM 8271, respectively, was that an increase in lipophilicity led to more active derivatives (Table 5).

In addition, those molecules were slightly more efficient against tested *Candida* spp. compared to the chosen Gram-positive and Gram-negative bacteria. The presence of the 3′-CF_3_ group was more beneficial than the substitution by the 4′-F atom. However, no compound from that series achieved the activity of simultaneously inspected standards, namely CPX, 5-FC, AMP or Amph. B (Table 5).

#### 2.2.2. Antiproliferative (Cytotoxicity) Screening

The compounds **5a**–**l** were subjected to the *in vitro* evaluation of their antiproliferative (cytotoxic) activity using the THP1-XBlue™-MD2-CD14 cell line derived from the human monocytic leukemia THP-1 cell line [39]. According to the estimated *IC*_50_ values, which expressed the concentration of a particular compound causing a 50% inhibition of a proliferation of the cell population, the derivatives have shown a non-antiproliferative (non-cytotoxic) potential with the *IC*_50_s > 10 μM.

### 2.3. Structure–Activity Relationships

The currently observed *MIC*s indicated that a comprehensive elucidation of the structure–antimicrobial activity relationships would be the most reasonable for the tested strains *M. kansasii* DSM 44162 and *M. marinum* CAMP 5644. As suggested in the previous sections of the paper, electronic (acidobasic) and steric parameters or the lipophilicity could notably affect the activity of the compounds **5a**–**l** against *M. kansasii*. To explore this hypothesis, experimentally observed p*K*_a_s [24,25], *in silico* generated *MV*s and experimental log *P*_exp_s [24,25,30], respectively, were correlated as the independent variables with the dependent variable(s), i.e., the biological data. In that analyses, antimycobacterial activities of inspected derivatives were expressed in the log (1/*MIC* [M]) units. The reference substances INH and RIF were excluded from the fitting analyses because it would not be correct to include their p*K*_a_s or the log *P*_exp_ outputs published in literature considering the very different experimental conditions of such estimations.

The data were fitted by using a linear function and a polynomial function of the 2^nd^ order, respectively. Adjusted *R*^2^ value, as the coefficient of a determination, or the coefficient of a multiple determination for a multiple regression, as a statistical measure of how close the data have been to the fitted regression line [40], was calculated for each analysis. The correlation coefficient *r* has been a relative measure of the quality of fit of a particular model because its value depended on the overall variance of the dependent variable [23]. In addition, the Fisher significance ratio (*F*), the residual sum of squares (*RSS*) data as well as the *Prob* > *F* values were calculated as the substantial statistical parameters. Those calculations were provided by the Origin Pro 9.0.0 software.

If the research concerned the electronic (acidobasic) properties of the evaluated compounds **5a**–**l**, the linear dependence between the p*K*_a_s and the log (1/*MIC* [M]) data was slightly preferred (*R*^2^ = 0.141) over the polynomial one (*R*^2^ = 0.070; Table S1), as shown in Figures S1 and S2 (Supplementary Materials). Equation (1) expressed the linear relationship between given descriptors, while the polynomial one was characterized by Equation (2) given below:

log (1/*MIC* [M]) = 5.970 (±1.262) − 0.337 (±0.201) × p*K*_a_ *r* = 0.375, *R*^2^ = 0.141, *F* = 2.80, *RSS* = 1.73, *Prob* > *F* = 0.125, *n* = 12 (1)

log (1/*MIC* [M]) = −1.795 (±16.039) + 2.140 (±5.103) × p*K*_a_ − 0.196 (±0.403) × (p*K*_a_)^2^ *r* = 0.265, *R*^2^ = 0.070, *F* = 1.41, *RSS* = 1.68, *Prob* > *F* = 1.413, *n* = 12 (2)

The relationship between the *MV*s, which were generated for the bases **5aB**–**lB**, and the log (1/*MIC* [M]) values was not linear nor polynomial (quasi-parabolic), as indicated the statistical parameters in Table S1 (Supplementary Materials).

Identically, the results from a linear regression analysis were completely unacceptable (Equation (S5) in Table S1 of Supplementary Materials) to characterize the relationship between the log *P*_exp_ outputs of all the studied compounds **5a**–**l** and the log (1/*MIC* [M])s. The polynomial (quasi‑parabolic) analysis provided slightly better (*R*^2^ = 0.035) but still not satisfactory results (Equation (S6) in Table S1 of Supplementary Materials).

All the inspected fitting models related to the *M. kansasii* strain were statistically characterized by the equations and the descriptors listed in Table S1. In addition, the relationships between the independent and the dependent variables were shown in Figures S1–S6 (Supplementary Materials).

The current research also revealed that there was a non-linear correlation between the p*K*_a_s and the log *P*_exp_ values. For clarification, the linear regression fitting provided *R*^2^ = 0.193 (Figure S7 in Supplementary Materials). Based on that knowledge, it was possible to perform a bilinear regression analysis to find a possible relationship between the p*K*_a_s, log *P*_exp_s and the log (1/*MIC* [M]) parameters. That dependence was described by Equation (3) given below:

log (1/*MIC* [M]) = 6.666 (±4.037) − 0.154 (±0.844) × log *P*_exp_ − 0.360 (±0.247) × p*K*_a_ *r* = 0.221, *R*^2^ = 0.049, *F* = 1.28, *RSS* = 1.72, *Prob* > *F* = 0.323, *n* = 12 (3)

Interesting findings resulted from the inspection of a relationship between the log *P*_exp_s of the 2- and 4-alkoxy substituted compounds and the log (1/*MIC* [M]) data. The observed quasi-parabolic dependence could be expressed by Equation (4) and was shown in Figure S8 (Supplementary Materials):

log (1/*MIC* [M]) = −61.556 (±32.636) + 36.145 (±18.220) × log *P*_exp_ − 4.995 (±2.540) × (log *P*_exp_)^2^ *r* = 0.488, *R*^2^ = 0.238, *F* = 2.09, *RSS* = 0.40, *Prob* > *F* = 0.219, *n* = 8 (4)

The linear relationship between the log *P*_exp_s and the log (1/*MIC* [M]) parameters of the 2-alkoxy substituted molecules (*R*^2^ = 0.973) was also found, as proven by Equation (5). Despite the main limitation of this investigation, a low *n* value, that knowledge could be very beneficial for further design of novel antimycobacterials containing the *N*-arylpiperazine pharmacophore.

log(1/*MIC* [M]) = 7.642 (±0.368) − 1.049 (±0.100) × log *P*_exp_ *r* = 0.986, *R*^2^ = 0.973, *F* = 109.84, *RSS* = 0.001, *Prob* > *F* = 0.009, *n* = 4 (5)

If the attention was turned to the *M. marinum* strain, a linear regression analysis of the p*K*_a_s and the log (1/*MIC* [M]) readouts provided more promising results, Equation (6) and Figure S9 (Supplementary Materials), than the linear fitting between the p*K*_a_ values and the activity against *M. kansasii*, expressed by the Equation (1):

log (1/*MIC* [M]) = 5.564 (±0.557) − 0.292 (±0.089) × p*K*_a_ *r* = 0.686, *R*^2^ = 0.471, *F* = 10.79, *RSS* = 0.34, *Prob > F* = 0.008, *n* = 12 (6)

The linear regression analysis of the *MV*s and the log (1/*MIC* [M]) data provided the results, which were very close to the outputs observed by a polynomial fitting model (Equations (S9) and (S10) in Table S2 of Supplementary Materials).

Very interesting values were obtained when the relationship between the *MV*s of the 3-alkoxy substituted derivatives and their log (1/*MIC* [M]) data based on the screening against *M. marinum* was examined by a linear function, as noted by Equation (7). In fact, a low number of the cases *n* = 4 could be regarded as an inconvenience for that approach:

log (1/*MIC* [M]) = 5.564 (±0.557) − 0.292 (±0.089) × *MV* *r* = 0.765, *R*^2^ = 0.585, *F* = 5.22, *RSS* = 0.024, *Prob > F* = 0.150, *n* = 4. (7)

The correlation of the log *P*_exp_s and the log (1/*MIC* [M]) values (*M. marinum*) provided a quasi-parabolic course (Figure S10 in Supplementary Materials), which was more convenient than the linear one (Equations (S11) and (S12) in Table S2 of Supplementary Materials). Moreover, the values of the statistical descriptors, which characterized that polynomial dependence, were better suited than the outputs, which resulted from a polynomial fitting of the log *P*_exp_s and the log (1/*MIC* [M]) data related to the *M. kansasii* strain (Equation (6) *versus* Equation (12) in Tables S2 and S3 of Supplementary Materials).

If the study was focused on the relationship between the lipophilicity of the 2- and 3-alkoxy substituted compounds and their activity against *M. marinum* (Figure S11 in Supplementary Materials), the statistical descriptors and Equation (8) indicated a clear polynomial dependence:

log (1/*MIC* [M]) = −36.615 (±4.208) + 22.594 (±2.361) × log *P*_exp_ − 3.151 (±0.331) × (log *P*_exp_)^2^ *r* = 0.963, *R*^2^ = 0.928, *F* = 46.32, *RSS* = 0.01, *Prob > F* = 6 ×10^−4^, *n* = 8 (8)

## 3. Discussion

### 3.1. Electronic, Steric and Lipohydrophilic Properties of the Compounds ***5a**–**l***

At the beginning, it was assumed that possible differences in the *in vitro* antimicrobial activity of the compounds **5a**–**l** could be caused by electronic, steric and lipohydrophilic properties of the substituents, which were attached to the salt-forming fragment (the 3′-CF_3_ group *versus* the 4′-F substituent) and the phenylcarbamoyloxy moiety (2-, 3- or 4-alkoxy side chain).

For a more comprehensive analysis of the results, the presence of the 3′-CF_3_ or 4′-F substituent was the principal factor according to which the compounds were divided into the Group I (**5a**–**f**) or II (**5g**–**l**), respectively. The strongly electron-withdrawing 3′-CF_3_ moiety has been regarded as bulkier and more lipophilic compared to the 4′-F atom, which has shown only moderate electron-withdrawing effect towards aromatic ring [23].

The expression of compounds’ electronic properties by the Hammett *σ* constant could be more complicated for the 2-substituted derivatives (**5a**, **5b**, **5g** and **5h**, respectively) because their *σ* values include a steric contribution. In general, compared to 3- or 4-substituents, the 2-ones could cause conformational changes, sometimes being favorable for interactions and sometimes being very unfavorable [23]. On those grounds, the p*K*_a_s of studied compounds [24,25] were involved in a more specific characterization of their electronic (as well as acidobasic) properties (Table 1).

Steric features of the non-protonated bases **5aB**–**lB** were described by the molecular volume (*MV*) values, which were generated *in silico* by an interactive Molecular Properties Calculator applet. As expected, the presence of the 3′-CF_3_ group with a simultaneous elongation of the alkoxy side chain led to higher *MV*s for the compounds **5a**–**f** compared to the values calculated for the **5g**–**l** set. Furthermore, the 2-substituted derivatives **5aB** and **5bB** were considered sterically bulkier than the 3- or 4-substituted ones (Table 1). However, a main disadvantage of that applet was the impossibility to generate relevant details for the biologically tested salts **5a**–**l**.

Compounds′ lipophilicity plays a notable role in their passage through a mycobacterial cell wall, which contains a large amount of lipid components [41]. It was confirmed experimentally [24,25,30] and *in silico* that all the inspected molecules **5a**–**l** were highly lipophilic (Table 2). The validity of performed log *P* calculations was checked for the atomic/fragmental and whole-molecule based methods *via* experimental log *P*_exp_s by the *F* and *AARS* values. The majority of predictive procedures were convenient (log *P*_Vf_, log *P*_Cf_ and CLOGP 4.0), excluding the ALOGPs (regarded as disputable) and the log *P*_Bf_ method (unacceptable). Following the values of the *F* and *AARS* descriptors, the attempt to calculate the log *P*s for the protonated forms of studied derivatives by the ALOGPs, not considering chloride anions in the calculations, was incorrect.

**Figure 3:**
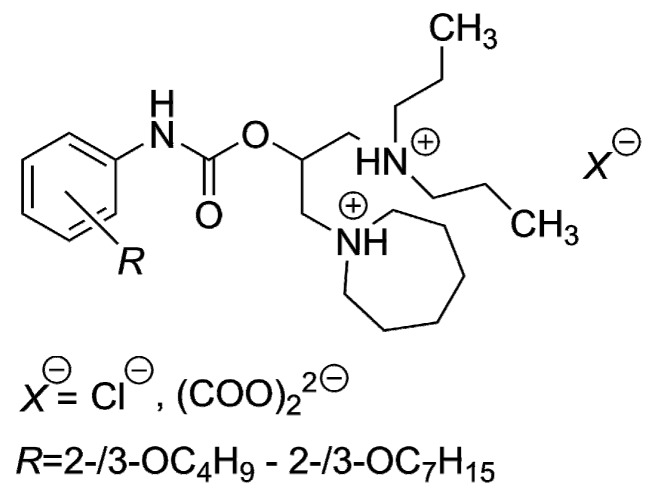
Dibasic esters of *R*-substituted phenylcarbamic acid previously inspected *in silico*.

The CLOGP approach has been applied in comprehensive (quantitative) structure–antimicrobial activity analyses very frequently [42,43,44,45,46,47,48] to predict the efficiency of molecules. The current research revealed that this well-known method could be altered by other *in silico* procedures, Ghose and Crippen′s (log *P*_Cf_) and Viswanadhan’s approach (log *P*_Vf_) might be good choices (Table 3). In addition, that CLOGP approach was not suitable for the lipophilicity prediction of structurally similar compounds [49], dibasic esters of *R*-substituted phenylcarbamic acid, which contained longer alkoxy side chains consisting of more than four carbon atoms (Figure 3). Moreover, the main disadvantage of the substructure methods was that the algorithms, according to which the log *P*s were generated, did not consider the position of the substituent [49].

### 3.2. Biological Assays in vitro

#### 3.2.1. Antimicrobial Susceptibility Testing

On the basis of the estimated *MIC*s, lipophilic molecules **5a**–**l** were more effective against *M. kansasii* DSM 44162 and *M. marinum* CAMP 5644 compared to *M. avium* subsp. *paratuberculosis* CIT03, *M. smegmatis* ATCC 700084, tested Gram-positive and Gram-negative bacteria or the yeasts from several *Candida* spp. (Table 4 and Table 5).

Among the members of the Group I, 1-[3-(3-ethoxyphenylcarbamoyl)oxy-2-hydroxypropyl]-4-(3-trifluoromethylphenyl)piperazin-1-ium chloride (**5d**) has shown a comparable activity against *M. kansasii* as the reference drug INH, while slightly lower activity was observed for 1-{2-hydroxy-3-(4-methoxyphenylcarbamoyl)oxy)propyl}-4-(3-trifluoromethylphenyl)piperazin-1-ium chloride (**5e**). Notable efficiency was observed for 1-[3-(3-ethoxyphenylcarbamoyl)oxy-2-hydroxypropyl]-4-(4-fluorophenyl)piperazin-1-ium chloride (**5j**), which was the most effective substance of both examined Groups I and II. In addition, the substance **5e** has shown the most promising potential to fight against *M. marinum*.

The study found out that electronic, steric and lipohydrophilic properties of the compounds **5a**–**l** influenced their effectiveness against tested strains of *M. kansasii* and *M. marinum* in a different manner (Table 4). Members of the genus *Mycobacterium* have been characterized by the presence of cell envelopes rich in unusual free lipids, interacting with a covalently anchored mycolyl-arabinogalactan-peptidoglycan matrix, which has formed an effective external permeability barrier [50]. Lipomannan and lipoarabinomannan, major mycobacterial cell wall lipoglycans of *M. kansasii*, have been considered important virulence factors as they modulate a host immune response [51]. Similarly, *M. marinum* produces large amounts of diacylglycosylphenolphthiocerol, a lipophilic “phenolic” glycolipid [52,53].

It could be suggested that some of investigated compounds, which have shown “a required level” of the lipophilicity (compounds **5d**, **5e** and **5j**, respectively), perturbated the biological membranes more easily and interacted with the complementary lipophilic structures. It could be also hypothesized that the fluorine atoms in the chemical structure of the Group I compounds interacted unspecifically with some mycobacterial effector sites. These effects could be based on the fluorines′ lipophilicity or the dipole–dipole interactions would be taken into consideration. Due to the presence of lone electron pairs, fluorine might increase a binding affinity towards certain complementary fragments of the mycobacteria. Such an increase would be more pronounced among the Group I derivatives. In the structure of the Group II compounds, one lone electron pair of the 4′-F substituent was involved in a conjugation with aromatic system.

**Figure 4:**
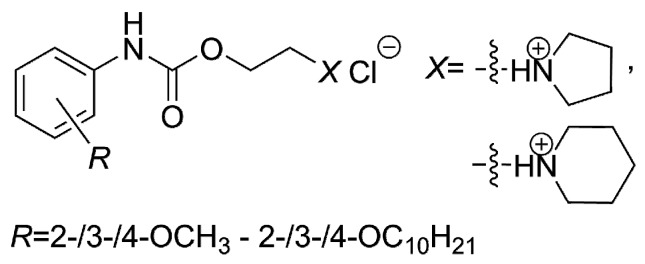
The *R*-substituted phenylcarbamic acid derivatives screened *in vitro* against *M. tuberculosis* H_37_R_v_, *M. kansasii* CNCTC My 235/80, *M. avium* CNCTC My 330/88 and *M. kansasii* 6509/96.

Moreover, it was quite questionable to consider (relatively high) lipophilicity absolutely crucial for the efficiency against both mycobacteria. Similar doubts were formulated in the research [54], which was focused on the *in vitro* activity of structurally similar compounds, the derivatives of phenylcarbamic acid containing a piperidin-1-yl or pyrrolidin-1-yl moiety as a salt-forming fragment (Figure 4), against *M. tuberculosis* H_37_R_v_, *M. kansasii* CNCTC My 235/80, *M. avium* CNCTC My 330/88 and *M. kansasii* 6509/96, respectively.

It was concluded that the proportional increase in activity to the lipophilicity was limited [54]. That dependence was described as the cut-off effect and it was systematically reviewed by Balgavý and Devínsky [55].

More comprehensive findings and conclusions, which resulted from the current research, were provided in further sections of the paper dealing with the structure–antimicrobial relationships.

Different electronic and steric properties or the increase in lipophilicity of the *R*^1^ and *R*^2^ substituents (Table 1 and Table 2) did not result in the discovery of very promising compounds against *Staphylococcus aureus* ATCC 29213, methicillin-resistant *S. aureus* 63718, *Escherichia coli* ATCC 25922, *Enterococcus faecalis* ATCC 29212, *Candida albicans* CCM 8261, *C. parapsilosis* CCM 8260 or *C. krusei* CCM 8271 (Table 5). It seemed that the pharmacophore of the derivatives **5a**–**l** or the choice of the *R*^1^ or *R*^2^ substituents was not probably optimal for the interaction with targeted effector sites of the mentioned microbial strains. It was recognized that none of those compounds has shown the *MIC* < 253.99 μM (Table 5).

#### 3.2.2. Antiproliferative (Cytotoxicity) Screening

The current research revealed that the molecules **5a**–**l**, which were relatively effective against *M. kansasii* DSM 44162, have also shown an insignificant antiproliferative (i.e., non-cytotoxic) impact on the THP1-XBlue™-MD2-CD14 cell line derived from the human monocytic leukemia THP-1 cell line. The statement was proven by the observed *IC*_50_ values > 10 μM for all the derivatives under the study, regardless of the electronic, steric and lipohydrophilic features of both *R*^1^ and *R*^2^ substituents. Only the compounds with the *IC*_50_s < 10 μM could be consider antiproliferative (cytotoxic) agents [39].

Gonec *et al.* [56] and Kauerova *et al.* [57] concluded that *in vitro* antiproliferative (cytotoxic) effects of the substituted hydroxynaphthanilides, which contained alkoxy- (where alkoxy = methoxy to butoxy, prop-2-yloxy, but-2-yloxy) or nitrophenylaminocarbonyl moieties, was connected with the position 3 (alkoxy derivatives) or 3 and 4 (nitro derivatives) of those substituents on aromatic ring. The elongation and branching of the 3-alkoxy chain, i.e., the increase in steric bulkiness and lipophilicity, or the introduction of the 4-NO_2_ group led to compounds, which could be used as model structures for the development of novel anticancer drugs [56,57]. The intensity of the described antiproliferative effect of the 3-/4-NO_2_ substituted substances [57] was related to: (i) the presence of the hydroxynaphthanilide pharmacophore; (ii) the linearity of the compounds and (iii) a strong electron-withdrawing influence of the NO_2_ group. Those electronic effects would be expressed by the value of *σ*_NO2_ = 0.71 (the position 3) and 0.78 (the position 4), respectively [23].

On the contrary, the alkoxy substituents attached to aromatic system (Table 1) have shown very weak electron-withdrawing or moderate electron-donating properties. If considering the OCH_3_ moiety, the *σ* value of 0.12 and −0.27 was related to its 3- and 4-position [23]. Similar readouts were published for the OC_2_H_5_ group [23] in the position 3 (0.10) or 4 (−0.24). In addition, the values of steric descriptors *L* and *B*_i_–*B*_iv_ proved that both OCH_3_ and OC_2_H_5_ substituents were sterically bulkier than the NO_2_ group [23].

**Figure 5:**
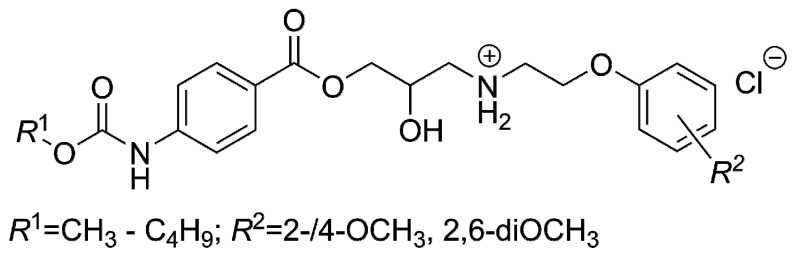
Chemical structure of 2-hydroxy-3-[(2-aryloxyethyl)amino]propyl-4-[(alkoxycarbonyl)amino]benzoates, which were efficient *in vitro* against *M. avium* subsp. *paratuberculosis* CIT03 strain.

Isosteric replacement of a 4-alkoxy side chain by the 4-alkoxycarbonylamino fragment and the modifications of both polar and salt-forming moieties led to the antimycobacterials (Figure 5), which have shown no antiproliferative (i.e., non-cytotoxic) impact on the THP-1 cell line regardless of the length of the *R*^1^ substituent or the *R*^2^ group(s) selection [58].

### 3.3. Structure–Activity Relationships

To provide a more comprehensive insight into the *SAR* relationships, the study suggested to consider experimentally observed p*K*_a_s [24,25], calculated *MV*s or experimentally estimated log *P*_exp_s [24,25,30] of the compounds **5a**–**l** important factors, which would mainly influence their activity against *M. kansasii* and *M. marinum* under the *in vitro* conditions.

Regarding the *M. kansasii* strain, the results indicated that a linear relationship between observed p*K*_a_s and the log (1/*MIC* [M]) values was slightly preferred over a quasi-parabolic relationship (Equations (1) and (2)). However, it was not possible to definitely confirm that lower p*K*_a_(s) meant more potent derivatives, especially due to the fact that the most effective substance **5j** (*MIC* = 17.62 μM), has shown the p*K*_a_ = 6.31. For the second most active molecule **5d** (*MIC* = 31.75 μM), the value of the p*K*_a_ = 5.35 was determined. Those electronic (acidobasic) properties seemed to be slightly more important for the efficiency of all the examined compounds **5a**–**l** compared to the steric or lipophilic ones.

The studies [59,60,61] concluded that electronic (acidobasic) features of structurally different compounds notably influenced their antimicrobial effectiveness. Higher *in vitro* activity of variously substituted pyrazinamides against *M. tuberculosis* H_37_R_v_ was connected with their relatively higher p*K*_a_s, which were calculated *in silico*. Those values led to lower ionizability of those pyrazinamide derivatives and their higher penetration to the cell [59]. In the case of diarylquinolines, which contained variously substituted *N*-alkylpiperazin-1-yl moiety, higher calculated basicity (p*K*_a_ > 8) supported their potency against *M. smegmatis* [60]. The research [61] confirmed a linear/bilinear relationship between the p*K*_a_ values of substituted sulfonamides and their activity against some Gram-positive and Gram-negative bacterial strains. Moreover, it was concluded [61], that the lipophilic properties of the sulfonamides, which were expressed by the log *k*’, log *P*_exp_ and *π* descriptors, were of a minor importance for their *in vitro* antibacterial efficiency.

The current research suggested that a presence of the *R*^1^ substituent attached to the 3-position, which would be sterically bulky, could lead to more active derivatives against *M. kansasii*. However, a more extensive set of the 3-substituted compounds would need to be examined in planned experiments to unequivocally verify this hypothesis. Furthermore, higher steric volume of the *R*^2^ group (*R*^2^ = 3′-CF_3_) was favorable for a potentiation of the activity of the 4-alkoxy substituted compounds against the *mycobacterium.* In fact, a crucial requirement was a presence of the short alkoxy chain (*R*^1^ = 4-methoxy).

At first sight, the *R*^2^, *r*, *RSS* or *F* values, which characterized both linear and quasi-parabolic relationships between the log *P*_exp_s of the series **5a**–**l** and their log (1/*MIC* [M]) data based on the *in vitro* screening against *M. kansasii*, were not very encouraging. The present analysis has shown that the increase in lipophilicity of the 2-alkoxy substituted compounds led to the decrease in activity. However, a limiting factor for that observation was the low number of investigated derivatives (*n* = 4). If the research was focused on the relationship between the log *P*_exp_s of the 2- and 4-alkoxy substituted compounds (*n* = 8) and their log (1/*MIC* [M])s, the observed dependence was very close to the cut-off effect. The maximum of potency was achieved when the compound has shown the log *P*_exp_ of approximately 3.60.

Considering the conclusions [55], several possible explanations of that phenomenon could be suggested for the presently screened derivatives. Firstly, the effect could be caused by the decrease in an achievable compound′s concentration at the site of action due to limited solubility. The drug′s partition coefficient between aqueous solution and the site of action increased less rapidly (with the increase in lipophilicity) than the aqueous solubility decreased, until a certain point. At that level, the maximum of an achievable concentration at the site of action was notably lower than that required to cause the biological effect. Secondly, the physicochemical properties (the lipophilicity) of the evaluated derivatives could suddenly change at a certain length of the alkoxy side chain or due to the position on a “core scaffold”, resulting in different types of interactions with the concerned site of the action. A third possibility, namely a perturbation of a membrane structure (a cell wall or a specific site, for example), which was suggested by Richards *et al.* [62], would not be very probable because of the short alkoxy side chain (methoxy or ethoxy) attached to aromatic system.

The present study also suggested that the electronic (acidobasic) features of the substances **5a**–**l** were more important for their efficiency against *M. marinum* than against *M. kansasii*. This statement was supported by the linear regression analyses and the statistical descriptors resulting from the corresponding equations (Tables S1 and S2 in Supplementary Materials). It was recognized that the compounds with the p*K*_a_ < 6.00 have shown stronger potential to be effective antimycobacterials. The value of the p*K*_a_ = 5.66 (Table 1) was estimated for the most active derivative **5e** (*MIC* = 65.32 μM).

Previous analysis [63], which was focused on charge-transfer complexes formed by ethyl-2-/3-/4-methoxyphenylcarbamates, has shown that the capability to release the electrons of aromatic ring to the partner *π*-electron acceptors was ranked in the order: 4-OCH_3_ derivative > 2‑OCH_3_ derivative > 3-OCH_3_ derivative. Those molecules could serve as simplified models of the currently analyzed compounds.

The present research suggested a possible interaction of the 4-alkoxyphenylcarbamoyloxy fragment (compound **5e** would be the best example) with appropriate effector sites of *M. marinum*, which have shown *π*-electron accepting properties, i.e., the interactions with some (hetero)aromatic moieties or systems containing double bonds. When the attention was paid to the 2-OCH_3_-substituted derivatives, a positive mesomeric effect of the side chain prevailed over the steric one, as published in the research [63]. Possible distorsion of the 2-methoxyphenylcarbamoyloxy moiety described by Mourad [63] was not regarded as inconvenient in the light of currently determined *MIC*s and their comparison to the *MIC* values, which were estimated for the 3-OCH_3_ substituted compounds **5c** and **5i**. That distorsion probably occurred in the structures of the compounds **5a** and **5g** due to a hydrogen bond formation between the oxygen of the substituent group and the hydrogen of a related carbamoyloxy fragment, leading to the formation of a five-membered ring, as noted in the paper [64].

Maximum efficiency against *M. marinum* was achieved if the compound has shown the log *P*_exp_ value of approximately 3.60. It seemed that the presence of the 2- or 3-alkoxy side chain would favor the lipophilic properties of the investigated molecules up to a certain level. On the contrary, the electronic features of the 4-alkoxy substituted derivatives played a more notable role, assuming “their certain length and the lipophilicity”, which could be ensured by a proper selection of both substituents *R*^1^ and *R*^2^. It was revealed that the introduction of *R*^1^ = 4-OCH_3_ and *R*^2^ = 3′-CF_3_ was the most convenient combination.

## 4. Materials and Methods

### 4.1. Tested Compounds

The compounds under current study **5a**–**l** were synthesized according to the Schemes S1 and S2 [30,65]. The details about synthetic procedures of the intermediates and final derivatives were given in the Supplementary Materials. Experimentally observed p*K*_a_ values (Table 1) and the log *P*_exp_s (Table 2) related to final compounds were published previously [24,25,30]. Other analytical grade compounds, which were used in the *in vitro* experiments as the standard drugs, were purchased as follows: isoniazid (INH), rifampicin (RIF), ciprofloxacin (CPX) from Sigma-Aldrich (Darmstadt, Germany), ampicillin (AMP), 5-flucytosine (5-FC) and amphotericin B (Amph. B) from Fluka Chemie (Buchs, Switzerland), respectively.

### 4.2. In silico Investigation

#### 4.2.1. Calculation of Molecular Volume

The molecular volume (*MV*) data (Table 1) were calculated using an interactive Molecular Properties Calculator applet (MolSoft LLC, San Diego, CA, USA).

#### 4.2.2. Prediction of Lipohydrophilic Properties

The values of the logarithm of a partition coefficient related to non-protonated basic molecules **5aB**–**lB** and to the reference drugs (Table 2) were calculated *in silico* for the octan-1-ol/water partitioning system (log *P*s) using the ChemBioDraw Ultra 12 software package (CambridgeSoft, Cambridge, MA, USA). The software provided fragmental CLOGP 4.0 [31], atomic Ghose and Crippen’s log *P*_Cf_ [32,33,34], atomic Viswanadhan′s log *P*_Vf_ [35] and atomic Broto′s log *P*_Bf_ [36] procedures. In addition, a whole-molecule ALOGPs [37] method, as an integral part of the Virtual Computational Chemistry Laboratory applet [66], was involved in the calculations as well. When the possibility to predict the values of the partition coefficients for biologically tested molecules **5a**–**l** was investigated, those methods could not correctly take into account their protonated forms (excluding the ALOGPs procedure) as well as stereochemical aspects (the presence of a stereogenic centre).

### 4.3. Antimicrobial Susceptibility Testing in vitro

The investigated compounds **5a**–**l** were *in vitro* screened as racemates against the chosen mycobacterial strains, Gram-positive, Gram-negative bacteria and against some yeasts.

#### 4.3.1. Antimycobacterial Evaluation

The *Mycobacterium avium* subsp. *paratuberculosis* CIT03 strain was grown in the Middlebrook (MB) broth, supplemented with the Oleic-Albumin-Dextrose-Catalase Supplement (OADC, Becton Dickinson, Oxford, UK) and the mycobactin J (2 μg/mL). The commercially available siderophore mycobactin J reduced the incubation period of *M. avium* subsp. *paratuberculosis* in a complex medium when compared to the medium containing the mycobactin P as a growth factor.

The identification of the isolate was performed using biochemical and molecular protocols. At a log phase growth, a culture sample (10 mL) was centrifuged at 15,000 rpm/20 min using a bench top centrifuge (Model CR 4-12, Jouan Inc., London, UK). Following removal of the supernatant, the pellet was washed in fresh Middlebrook 7H9GC broth and re-suspended in fresh supplemented MB broth (10 mL). The turbidity was adjusted to match the McFarland standard No. 1 containing approximately 3.0 × 10^8^ Colony Forming Units (CFU) with the MB broth. Further 1:20 dilution of the culture was performed in the MB broth. The susceptibility of given mycobacterial species was investigated in a 96-well plate format. In those experiments, sterile deionised water (300 μL) was added to all outer-perimeter wells of the plates to minimize an evaporation of the medium in the test wells during the incubation process. Each evaluated compound (100 μL) was incubated with the mycobacterial species (100 μL). The dilutions of a particular compound were prepared in triplicate. For all inspected derivatives, final concentrations ranged from 1000 μg/mL to 60 μg/mL. All compounds were prepared in dimethyl sulfoxide (DMSO; Sigma-Aldrich, Irvine, UK) and subsequent dilutions were made in the supplemented MB broth. The plates were sealed with a parafilm and incubated at 37 °C for 11 days. Following the incubation, a 10% addition of a water‑soluble dye, the alamarBlue reagent (AbD Serotec, Kidlington, UK), was mixed into each well. Absorbance readings at 570 nm and 600 nm were taken, initially for background subtraction and after 24 h re-incubation. The subtraction is necessary for strongly coloured compounds, where the colour may interfere with the interpretation of any colour change. For non-interfering compounds, a blue colour in a well was interpreted as an absence of a growth and a pink colour was scored as a growth.

The minimum inhibitory concentration (*MIC*) was defined as the lowest concentration of the compound at which no visible bacterial growth was observed. In other words, the *MIC* was the lowest concentration that prevented a visual colour change from blue to pink. The *MIC* value has been routinely and widely used in bacterial assays and it has been a standard detection limit according to the Clinical and Laboratory Standards Institute [67,68]. The clinically used antimycobacterial drugs INH and RIF were applied as the standards. The estimated *MIC*s, expressed in μM units, were summarized in Table 4.

The *in vitro* screening of the compounds **5a**–**l** was also performed against *M. smegmatis* ATCC 700084, *M. kansasii* DSM 44162 as well as *M. marinum* CAMP 5644. The broth dilution micromethod in the Middlebrook 7H9 medium (Difco, Lawrence, MO, USA) supplemented with the BD BBL™ Middlebrook ADC Enrichment (Becton, Dickinson & Company, Franklin Lakes, NJ, USA) was used to determine the minimum inhibitory concentration (*MIC*), as described in the article [69].

The tested molecules **5a**–**l** and the standard drugs were dissolved in DMSO (Sigma-Aldrich) and the final concentration of DMSO did not exceed 2.5% of the total solution composition. Those final concentrations, ranging from 256 μg/mL to 0.125 μg/mL, were obtained by a two-fold serial dilution of the stock solution in a microtiter plate with a sterile medium.

Bacterial inocula were prepared by transferring the colonies from culture into sterile water. The cell density was adjusted to the 0.5 McFarland units using the Densi-La-Meter (LIAP, Riga, Latvia). The final inoculum was made by a 1:1000 dilution of the suspension with sterile water. Drug-free controls, sterility controls and the controls consisted of a medium and DMSO alone were included. The results were determined visually after static incubation in the darkness in an aerobic atmosphere for: (i) 3 days at 37 °C for *M. smegmatis*; (ii) 7 days at 37 °C for *M. kansasii* and (iii) 21 days at 28 °C for *M. marinum*.

The *MIC* was defined as the lowest concentration of the compound at which no visible bacterial growth was observed. The *MIC* value has been routinely and widely used in bacterial assays and it has been considered a standard detection limit according to the Clinical and Laboratory Standards Institute [67,68]. The reference drugs INH and RIF were simultaneously inspected *in vitro*. The observed *MIC*s in μM units were summarized in Table 4.

#### 4.3.2. Antibacterial Evaluation

The synthesized compounds **5a**–**l** were also *in vitro* screened against Gram-positive bacterial strains, a clinical isolate of methicillin-resistant *Staphylococcus aureus* 63718 (abbreviation used: *MRSA* 63718) carrying the mecA gene [70], vancomycin-susceptible methicillin-susceptible *S. aureus* ATCC 29213 (*SA* 29213) and vancomycin-resistant *Enterococcus faecalis* ATCC 29212 (*VRE* 29212), specifically. The *MRSA* 63718 and *SA* 29213 strains were obtained from the National Institute of Public Health (Prague, Czech Republic), suspected colonies were confirmed by a polymerase chain reaction testing; a 108 bp fragment specific for *S. aureus* was detected [71]. The *VRE* 29212 strain was isolated from crows in Northern America [72]. The *Escherichia coli* ATCC 25922 (*EC* 25922) strain was used as a representative of Gram-negative bacteria. Prior to the testing, each strain was transferred into a nutrient agar (Oxoid Limited, Basingstoke, UK), which was enriched with 5% defibrinated bovine blood.

The *MIC*s were determined by the microtitration broth method in the cation-adjusted Mueller-Hinton broth (Becton Dickinson, Oxford, UK) according to an international interdisciplinary Clinical Laboratory Standard Institute guidelines [73,74].

Brain-Hearth Infusion agar (Oxoid Limited, Basingstoke, UK) was used as a recommended medium for the agar screen susceptibility testing of *VRE* 29212 [75]. The compounds **5a**–**l** and the reference substances (AMP and CPX) were dissolved in DMSO (Sigma-Aldrich, Darmstadt, Germany) and the final concentration of DMSO did not exceed 2.5% of the total solution composition. The final concentrations, ranging from 256 μg/mL to 0.125 μg/mL, were obtained by a two-fold serial dilution of the stock solutions in a microtitration checkerboard. The checkerboard was inoculated by the bacterial inocula, which were prepared in a phosphate buffered saline solution with the p*H* = 7.2–7.3 to simulate physiological p*H*. The final concentration of each inoculum in the final volume of 100 μL in the wells was approximately 7.5 × 10^6^ CFU/mL. Drug-free controls, sterility controls and the controls consisted of the broth with DMSO were included. The determination of the results was performed visually after 24 h of static incubation in the darkness at 37 °C in an aerobic atmosphere.

The *MIC* was defined as the lowest concentration of the compound at which no visible bacterial growth was observed [75]. To ensure reproducibility, each antibacterial *MIC* assay was performed triplicate on separate occasions. The *MIC*s (in μM units), as the averages of three estimations, were summarized in Table 5.

#### 4.3.3. Candidacidal Evaluation

In order to provide a look into the potential usefulness of the derivatives **5a**–**l** as the candidates for a development of anti-yeast (candidacidal) agents, they were *in vitro* tested against *Candida albicans* CCM 8261 (*CA* 8261), *C. krusei* CCM 8271 (*CK* 8271) and *C. parapsilosis* CCM 8260 (*CP* 8260). The yeasts were obtained from the Czech Collection of Microorganisms (Masaryk University, Faculty of Science, Brno, Czech Republic). They were grown and kept on Sabouraud agar (Oxoid Limited, Basingstoke, UK).

For the screening, the microdilution method according to the Antimicrobial Susceptibility Testing Protocols was applied [69]. The inoculum was prepared from the cultures grown on Sabouraud agar. With the sterile solution of NaCl, the inoculum was prepared to the 0.5 McFarland units using the Densi-La-Meter densitometer (LIAP, Riga, Latvia) and diluted a thousand‑fold with the Roswell Park Memorial Institute (RPMI) 1640 medium (Sigma-Aldrich, Darmstadt, Germany). Prepared suspension was used for an inoculation of microtitre plates. Final concentration in wells ranged from 5.0 × 10^2^ CFU/mL to 2.5 × 10^3^ CFU/mL. The compounds **5a**–**l** and the reference drugs (5-FC and Amph. B) were dissolved in DMSO (Sigma-Aldrich) and the final concentration of DMSO did not exceed 2.5% of the total solution composition. For the determination of the *MIC*s, the stock solutions of all the tested compounds were diluted in the microtitre checkerboard to the final concentration varying from 128 μg/mL to 0.125 μg/mL. Drug-free control, sterility control and the control consisted of a medium and DMSO alone were included. Inoculated microtitre plates were incubated for 48 h in the darkness at 37 °C in an aerobic atmosphere. The results were estimated against static light.

The *MIC* was defined as the lowest concentration of the tested compound at which no visible growth was observed. To ensure reproducibility of the results, each candidacidal *MIC* assay was performed triplicate on separate occasions. The *MIC*s (in μM units) were summarized in Table 5. It was important to note that all the *MIC*s from the *in vitro* antimicrobial screening were expressed in μg/mL units and were available in Tables S3 and S4 (Supplementary Materials).

### 4.4. Antiproliferative (Cytotoxicity) Screening in vitro

#### 4.4.1. Cell Culture

The THP1-XBlue™-MD2-CD14 cell line was obtained from InvivoGen (San Diego, CA, USA). The cell line was derived from the human monocytic leukemia THP-1 cell line. The cells were cultivated at 37 °C in the RPMI 1640 medium, which was supplemented with 2 mM l-glutamine (Biosera, Nuaillé, France), 10% heat-inactivated fetal bovine serum (HyClone, GE Healthcare, Logan, Utah, USA), 100 U/mL penicillin, and 100 μg/mL streptomycin (Biosera) in a humidified atmosphere containing 5% CO_2_. The culture was split twice a week, when the cells reached a concentration of 5.0 × 10^5^–7.0 × 10^5^ cells/mL.

#### 4.4.2. Analysis of Cell Proliferation and Viability

Cell number and viability were determined by following staining with erythrosin B solution. The solution, which consisted of 0.1% erythrosin B (*w*/*v*; Sigma-Aldrich, Darmstadt, Germany) in a phosphate buffered saline with the p*H* = 7.2–7.4, was mixed with an equal amount of the cell suspension and the numbers of viable and non-viable cells were counted using a hemocytometer and a light microscope. The cells that remained unstained were considered viable, the light red ones as non-viable.

The THP1-XBlue™-MD2-CD14 cells (5.0 × 10^5^ cells/mL) were transferred into a serum-free RPMI 1640 culture medium and seeded into the 96-well plates (100 μL/well) in triplicate. The measurements were taken 24 h after a treatment with increasing concentrations (0.12–10 μM) of the tested compounds **5a**–**l** dissolved in DMSO (Sigma-Aldrich). The maximum concentration of DMSO in the assays never exceeded 0.1%.

The viability was measured by the Cell Proliferation Reagent kit WST-1 (Roche Diagnostics, Basel, Switzerland), according to the manufacturer’s manual. In the assays, a stable sodium 4-(3-(4-iodophenyl)-2-(4-nitrophenyl)-2,3-dihydro-1*H*-tetrazol-3-ium-5-yl)benzene-1,3-disulfonate (a water soluble tetrazolium; WST-1) was cleaved to a soluble formazan by a complex cellular mechanism that occurred primarily at the cell surface. Given bioreduction was dependent on glycolytic production of NAD(P)H in viable cells, therefore, the amount of formazan dye formed directly correlated to the number of metabolically active cells in the culture. That amount was calculated as a percentage of the control cells, which were treated only with DMSO and were assigned as 100%. Solvent control was included to verify that the DMSO has shown no effect at the used concentration. The *IC*_50_ values were calculated according to the equation for Boltzman sigmoidal concentration–response curves. For the calculations, the GraphPad Prism 5.02 software (GraphPad Software Inc., La Jolla, CA, USA) was used, as published in the research article [39].

### 4.5. Statistical Analysis

The validity of the log *P* predictions was checked for employed *in silico* methods *via* experimental log *P*_exp_s for the compounds **5a**–**l** by the Fisher′s *F*-test (the *F* value) to express the differences between the experimental and the calculated values. Those calculations were performed by the Origin Pro 9.0.0 software (OriginLab Corporation, Northampton, MA, USA). In addition, the validity of five calculation procedures was compared as follows: the averaged absolute residual sums (*AARS*) for the differences between the experiment and the calculation were given as another statistical criterion. The differences (Δlog *P*) between the log *P*_exp_ and predicted data in the range of 0.00 to ±0.49 were qualified as acceptable, Δlog *P* values of ±0.50 to ±0.99 were viewed as disputable and differences exceeding ±0.99 were classified as unacceptable. The numbers of calculations exhibiting higher or lower values than the log *P*_exp_s were also counted [38], as listed in Table 3. The *AAR*S were calculated by the Microsoft Office Excel 2010 program (Microsoft Corporation, Redmond, WA, USA).

The regression equations, the *R*^2^, *r*, *RSS*, *F* and *Prob > F* statistical characteristics and the figures, which characterized the relationships between the independent variables (the p*K*_a_s, the *MV*s and the log *P*_exp_s) and the dependent variable(s), i.e., the *in vitro* antimicrobial activity expressed in the log (1/*MIC* [M]) units, were calculated and visualized by the Origin Pro 9.0.0 software.

## 5. Conclusions

Electronic (theoretical *σ* values, observed p*K*_a_s), steric (calculated *MV*s) and lipohydrophilic (experimentally determined log *P*_exp_s *versus* calculated log *P*s) properties of the *N*-arylpiperazine derivatives **5a**–**l** were investigated together with their *in vitro* biological activities. The biological evaluation consisted of the screening against various mycobacterial strains, Gram-positive, Gram-negative bacteria and the yeasts, respectively, and the inspection of an antiproliferative (cytotoxic) potential.

The molecules **5a**–**l** were considered to range from weakly acidic to neutral (p*K*_a_ = 5.35–7.24) and be highly lipophilic (log *P*_exp_ = 3.28–3.90). The *in silico* evaluation of their basic forms **5aB**–**lB** provided the *MV*s in the range from 405.33 Å^3^ to 436.81 Å^3^. It was also found that the CLOGP approach, a method very frequently involved in the prediction of the lipophilicity in complex structure–antimicrobial activity analyses, could be altered by atomic/fragmental Ghose and Crippen′s or Viswanadhan′s procedures for the inspected set. The screened compound 1-[3-(3-ethoxyphen-ylcarbamoyl)oxy-2-hydroxypropyl]-4-(3-trifluoromethylphenyl)piperazin-1-ium chloride (**5d**) has shown *MIC* = 31.75 μM against *M. kansasii* DSM 44162. Its potency was comparable to that of the INH standard (*MIC* = 29.17 μM). In addition, 1-[3-(3-ethoxyphenylcarbamoyl)oxy-2-hydroxypropyl]-4-(4-fluorophenyl)piperazin-1-ium chloride (**5j**) was even more active (*MIC* = 17.62 μM). Among the tested *N*-arylpiperazines, 1-{2-hydroxy-3-(4-methoxyphenylcarbamoyl)oxy)propyl}-4-(3-trifluoro-ethylphenyl)piperazin-1-ium chloride (**5e**) was the most efficient against *M. marinum* CAMP 5644 (*MIC* = 65.32 μM) and has also shown the *MIC* seven times lower than that of INH (*MIC* = 466.68 μM). On the other hand, the RIF reference drug was regarded as the most promising (*MIC* = 2.43 μM).

All the screened *N*-arylpiperazines have shown an insignificant antiproliferative effect, and could be regarded as non-cytotoxic. The research preliminary suggested that there was probably a very limited connection (if any) between the mechanism of antimycobacterial action of the studied substances and their ability to inhibit the cell proliferation process.

The electronic (acidobasic), steric and lipohydrophilic features of those molecules influenced their *in vitro* antimicrobial profile, especially the effectiveness against the two mycobacterial strains, *M. kansasii* and *M. marinum*. In the *SAR* analyses, the antimycobacterial activity was expressed in the log (1/*MIC* [M]) units.

In general, electronic properties (p*K*_a_) seemed to be slightly more important for the activity of the entire set **5a**–**l** against *M. kansasii* compared to the steric or lipophilic ones. It was not possible definitely confirm that the p*K*_a_s < 6.00 led to more potent derivatives. That “hypothesis” was valid for the compounds **5d** (p*K*_a_ = 5.35) and **5e** (p*K*_a_ = 5.66), however, the most active molecule **5j** has shown the p*K*_a_ = 6.31.

There was recognized neither linear nor quasi-parabolic relationship between the lipophilicity of tested molecules **5a**–**l** and their potency against mentioned *mycobacterium*. Considering the positional isomerism of the attached *R*^1^ group, a more detailed view revealed that the increase in lipophilicity of the 2-alkoxy substituted compounds led to the decrease in the efficiency. If the research was focused on the 2- and 4-alkoxy substituted derivatives, a cut-off effect, i.e., a quasi-parabolic dependence, could be observed. The elongation of the *R*^1^ substituent, thus the increase in lipophilicity, was favorable if attached to the 3-position. The maximum activity was achieved if the compound has shown the log *P*_exp_ value of about 3.60.

Regarding the *M. marinum* strain, the lower p*K*_a_s of the analyzed compounds **5a**–**l** (p*K*_a_ ≤ 6.00) provided a higher probability to fight more effectively against that *mycobacterium*. The increase in an electron density on aromatic ring of the phenylcarbamoyloxy moiety was more important for the activity of the 4-alkoxy substituted derivatives compared to an increase in lipophilicity.

The presence of a short alkoxy moiety (*R*^1^ = 4-methoxy) in the lipophilic part together with a sterically bulkier substituent *R*^2^ (*R*^2^ = 3′-CF_3_) attached to the phenyl ring within a salt-forming group would be the most convenient combination. The research suggested a possible interaction of the 4‑alkoxyphenylcarbamoyloxy fragments of the screened molecules with effector sites of *M. marinum*, which have shown *π*-electron accepting properties. Sterically bulkier *R*^1^ (in the 3-position) and *R*^2^ substituents were considered structural alternatives, which contributed to only a slight improvement in the activity.

Similarly to the *M. kansasii* strain, a quasi-parabolic dependence between the log *P*_exp_s of the 2- and 3-alkoxy substituted compounds and their activity against *M. marinum* was recognized. Concerning the whole series of inspected *N*-arylpiperazine derivatives, the most effective antimycobacterials have shown the log *P*_exp_ value of approximately 3.60. In conclusion, some of the *N*-arylpiperazines discussed above were found to have promising *in vitro* antimycobacterial potential and could be considered for further optimization to develop novel active agents.

## Figures and Tables

**Table 1:** Experimentally observed values of the dissociation constants (p*K*_a_) of the compounds **5a**–**l** and *in silico* generated molecular volume (*MV*) data.

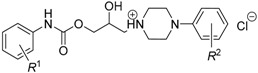				
Compound	*R*^1^	*R*^2^	p*K*_a_	*MV* [Å^3^] ^1^
**5a**	2-OCH_3_	3′-CF_3_	5.83	418.25
**5b**	2-OC_2_H_5_	3′-CF_3_	6.00	436.81
**5c**	3-OCH_3_	3′-CF_3_	5.73	417.93
**5d**	3-OC_2_H_5_	3′-CF_3_	5.35	436.49
**5e**	4-OCH_3_	3′-CF_3_	5.66	417.85
**5f**	4-OC_2_H_5_	3′-CF_3_	5.69	436.41
**5g**	2-OCH_3_	4′-F	6.73	387.16
**5h**	2-OC_2_H_5_	4′-F	6.58	405.73
**5i**	3-OCH_3_	4′-F	6.55	386.84
**5j**	3-OC_2_H_5_	4′-F	6.31	405.40
**5k**	4-OCH_3_	4′-F	7.24	386.77
**5l**	4-OC_2_H_5_	4′-F	7.18	405.33

^1^ *MV*, Molecular volume, the outputs were generated for non-protonated bases **5aB**–**lB** (Scheme S2 in Supplementary Materials).

**Table 2:** Experimentally observed values of the partition coefficient (log *P*_exp_) of the compounds **5a**–**l** in the octan-1-ol/phosphate buffer (p*H* = 7.4) system and *in silico* predicted readouts for corresponding non-protonated bases and the reference drugs isoniazid (INH), rifampicin (RIF), ciprofloxacin (CPX), 5-flucytosine (5-FC), ampicillin (AMP) and amphotericin B (Amph. B), respectively, by CLOGP method (CLOGP 4.0), Ghose and Crippen′s approach (log *P*_Cf_), Viswanadhan′s approach (log *P*_Vf_), Broto′s algorithm (log *P*_Bf_) and ALOGPs method.

Compound	log *P*_exp_	CLOGP 4.0	log *P*_Cf_	log *P*_Vf_	log *P*_Bf_	ALOGPs	
						*Base* ^3^	*Salt* ^4^
**5a**	3.57	4.21	3.62	3.71	2.52	3.12	2.36
**5b**	3.60	4.74	3.95	4.06	2.86	3.64	2.41
**5c**	3.61	4.21	3.62	3.71	2.52	3.14	2.35
**5d**	3.72	4.74	3.95	4.06	2.86	3.66	2.45
**5e**	3.60	4.21	3.62	3.71	2.52	3.17	2.35
**5f**	3.71	4.74	3.95	4.06	2.86	3.69	2.46
**5g**	3.61	3.34	2.85	2.97	1.67	2.24	2.52
**5h**	3.90	3.87	3.19	3.31	2.02	2.76	2.88
**5i**	3.25	3.34	2.85	2.97	1.67	2.31	2.52
**5j**	3.59	3.87	3.19	3.31	2.02	2.78	2.87
**5k**	3.42	3.34	2.85	2.97	1.67	2.37	2.54
**5l**	3.28	3.87	3.19	3.31	2.02	2.76	2.89
INH	*nd* ^1^	−0.67	−0.64	−0.44	−0.69	−0.71	*ng*
RIF	*nd*	4.03	*ng* ^2^	*ng*	*ng*	2.35	*ng*
CPX	*nd*	−0.73	1.32	1.59	*ng*	−0.57	*ng*
5-FC	*nd*	−1.63	−1.26	−1.18	−0.38	−0.24	*ng*
AMP	*nd*	−1.20	−0.20	0.14	*ng*	0.88	*ng*
Amph. B	*nd*	−3.65	*ng*	*ng*	*ng*	−0.66	*ng*

^1^ *nd*, Not experimentally determined; ^2^ *ng*, the computational data were not generated; ^3^ *Base*, calculated outputs for non-protonated bases **5aB**–**lB**; ^4^ *Salt*, calculated outputs for protonated salts **5a**–**l**.

**Table 3:** Comparative validity check of employed calculation programs.

Compound ^1^	CLOGP 4.0	log *P*_Cf_	log *P*_Vf_	log *P*_Bf_	ALOGPs	
					*Base* ^2^	*Salt* ^3^
*AARS*	−0.47	0.17	0.06	1.30	0.60	1.02
acceptable	5	9	10	0	6	1
disputable	4	3	2	3	2	2
unacceptable	3	0	0	9	3	8
>log *P*_exp_	9	6	7	12	11	0
<log *P*_exp_	3	6	5	0	1	12

^1^ Compound, the set of investigated derivatives **5a**–**l**; ^2^ *Base*, calculated outputs for non-protonated bases **5aB**–**lB**; ^3^ *Salt*, calculated outputs for protonated salts **5a**–**l**.

**Table 4:** Antimycobacterial screening *in vitro* (*MIC*) of the compounds **5a**–**l** compared to the isoniazid (INH) and rifampicin (RIF) standards.

Compound	*MIC* (μM)			
	*MAP* ^1^	*MS* ^2^	*MK* ^3^	*MM* ^4^
**5a**	510.29	522.53	130.63	130.63
**5b**	1984.32	507.99	127.00	127.00
**5c**	1020.57	522.53	130.63	130.63
**5d**	1984.32	**127.00**	**31.75**	127.00
**5e**	1020.57	522.53	**65.32**	**65.32**
**5f**	992.16	507.99	507.99	253.99
**5g**	2273.19	581.94	145.48	145.48
**5h**	1101.47	563.95	281.98	281.98
**5i**	1136.60	581.94	145.48	290.97
**5j**	550.73	281.98	**17.62**	140.99
**5k**	2273.19	581.94	290.97	581.94
**5l**	2202.93	563.95	563.95	281.98
INH	1822.95	117.03	29.17	466.68
RIF	109.36	19.40	0.15	2.43

^1^ *MAP*, *Mycobacterium avium* subsp. *paratuberculosis* CIT03; ^2^ *MS*, *M. smegmatis* ATCC 700084; ^3^ *MK*, *M. kansasii* DSM 44162; ^4^ *MM*, *M. marinum* CAMP 5644. The *N*-arylpiperazines, which have shown promising antimycobacterial activity, were highlighted by the bold font style in gray.

**Table 5:** The efficiency *in vitro* (*MIC*) of the compounds **5a**–**l** and the reference drugs ciprofloxacin (CPX), 5-flucytosine (5-FC), ampicillin (AMP) and amphotericin B (Amph. B), respectively, against chosen Gram-positive and Gram-negative bacterial strains and the yeasts.

Compound	*MIC* (μM)						
	*SA* ^1^	*MRSA* ^2^	*EC* ^3^	*EF* ^4^	*CA* ^5^	*CP* ^6^	*CK* ^7^
**5a**	>522.53	>522.53	>522.53	>522.53	>261.27	>261.27	>261.27
**5b**	>507.99	>507.99	>507.99	>507.99	>253.99	>253.99	>253.99
**5c**	>522.53	>522.53	>522.53	>522.53	>261.27	>261.27	>261.27
**5d**	>507.99	>507.99	>507.99	>507.99	>253.99	>253.99	>253.99
**5e**	>522.53	>522.53	>522.53	>522.53	>261.27	>261.27	>261.27
**5f**	>507.99	>507.99	>507.99	>507.99	>253.99	>253.99	>253.99
**5g**	>581.94	>581.94	>581.94	>581.94	>290.97	>290.97	>290.97
**5h**	>563.95	>563.95	>563.95	>563.95	>281.98	>281.98	>281.98
**5i**	>581.94	>581.94	>581.94	>581.94	>290.97	>290.97	>290.97
**5j**	>563.95	>563.95	>563.95	>563.95	>281.98	>281.98	>281.98
**5k**	>581.94	>581.94	>581.94	>581.94	>290.97	>290.97	>290.97
**5l**	>563.95	>563.95	>563.95	>563.95	>281.98	>281.98	>281.98
CPX	0.75	>48.29	>48.29	>48.29	*nd*	*nd*	*nd*
5-FC	*nd* ^8^	*nd*	*nd*	*nd*	7.69	61.50	0.96
AMP	5.72	>45.79	>45.79	>45.79	*nd*	*nd*	*nd*
Amph. B	*nd*	*nd*	*nd*	*nd*	0.54	1.08	0.54

^1^ *SA*, *Staphylococcus aureus* ATCC 29213; ^2^ *MRSA*, methicillin-resistant *Staphylococcus aureus* 63718; ^3^ *EC*, *Escherichia coli* ATCC 25922; ^4^ *EF*, *Enterococcus faecalis* ATCC 29212; ^5^ *CA*, *Candida albicans* CCM 8261; ^6^ *CP*, *Candida parapsilosis* CCM 8260; ^7^ *CK*, *Candida krusei* CCM 8271; ^8^ *nd*, not determined, the compound was not used as the standard in the experiment.

## Supplementary Materials

Supplementary materials can be accessed at: http://www.mdpi.com/1420-3049/21/10/1274/s1.

## Abbreviations

The following abbreviations are used in this manuscript:

5-FC	5-Flucytosine
*AARS*	Averaged absolute residual sums
AMP	Ampicillin
Amph. B	Amphotericin B
CPX	Ciprofloxacin
DMSO	Dimethyl sulfoxide
*F*	Fisher significance ratio
INH	Isoniazid
log *k*’	Capacity (retention) factor
log *P*_exp_	Experimentally observed values of a partition coefficient in the octan-1--ol/phosphate buffer (p*H* = 7.4) system
*MIC*	Minimum inhibitory concentration
*MV*	Molecular volume
*n*	Number of cases
PS(s)	Privileged substructure(s)
*r*	Correlation coefficient
*R*^2^	Coefficient of determination
RIF	Rifampicin
*RSS*	Residual sum of squares
*SAR*	Structure–activity relationship(s)
