# Supplementary material for: The Structure–Antimicrobial Activity Relationships of a Promising Class of the Compounds Containing the N-Arylpiperazine Scaffold
Source: Molecules. 2016 Sep 26;21(10):1274. doi: 10.3390/molecules21101274 (PMC6273431; doi:10.3390/molecules21101274)
Supplement: Supplementary file 1 [file molecules-21-01274-s001.pdf]

Supplementary Materials: The Structure–Antimicrobial Activity Relationships of a Promising Class of the Compounds Containing the *N*-Arylpiperazine Scaffold †

Ivan Malík, Jozef Csöllei, Josef Jampílek, Lukáš Stanzel, Iveta Zadražilová, Jan Hošek, Šárka Pospíšilová, Alois Čížek, Aidan Coffey and Jim O´Mahony

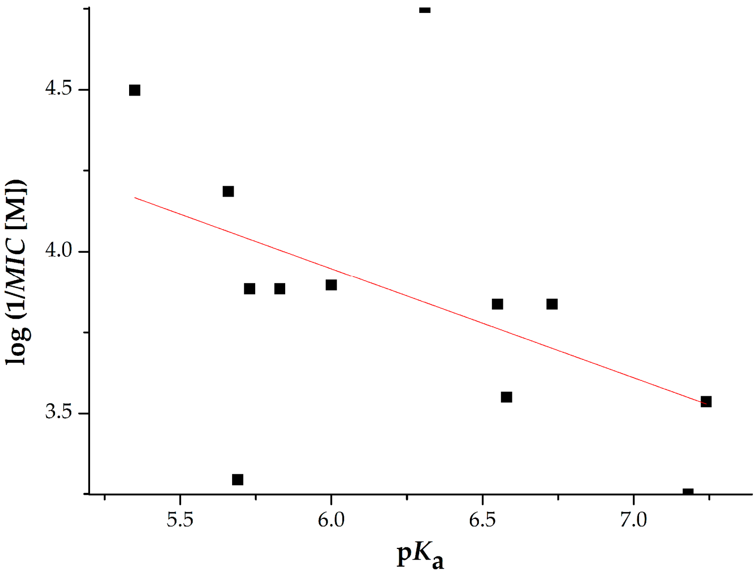

	Intercept Value	Intercept Standard Error	Slope Value	Slope Standard Error	Statistics Adj. R-Square
B	5.97022	1.26164	-0.33718	0.20135	0.14091

Figure S1. The relationship between the activity (in the log (1/*MIC* [M]) units) of the tested compounds **5a–l** against *M. kansasii* DSM 44162 and their electronic properties (pK_a) investigated by a linear fitting analysis model.

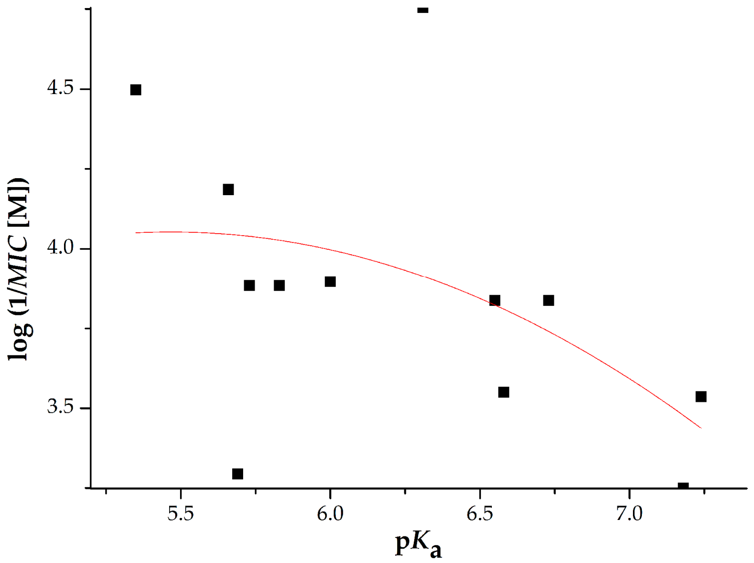

	Intercept	Intercept	B1	B1	B2	B2	Statistics
	Value	Standard Error	Value	Standard Error	Value	Standard Error	Adj. R-Square
B	-1.79474	16.0392	2.13955	5.10304	-0.19571	0.40289	0.06985

Figure S2. The relationship between the activity (in the log (1/*MIC* [M]) units) of the tested compounds **5a–l** against *M. kansasii* DSM 44162 and their electronic properties (pK_a) investigated by a polynomial fitting analysis model.

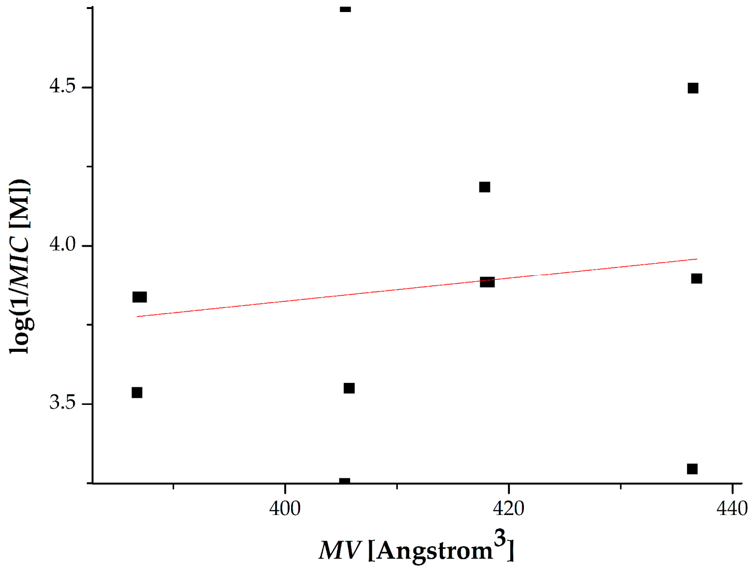

	Intercept	Intercept	Slope	Slope	Statistics
	Value	Standard Error	Value	Standard Error	Adj. R-Square
B	2.36311	3.05258	0.00365	0.00741	-0.07388

Figure S3. The relationship between the activity (in the log (1/*MIC* [M]) units) of the tested compounds **5a–l** against *M. kansasii* DSM 44162 and the molecular volume (*MV*) data of particular bases **5aB–lB** investigated by a linear fitting analysis model.

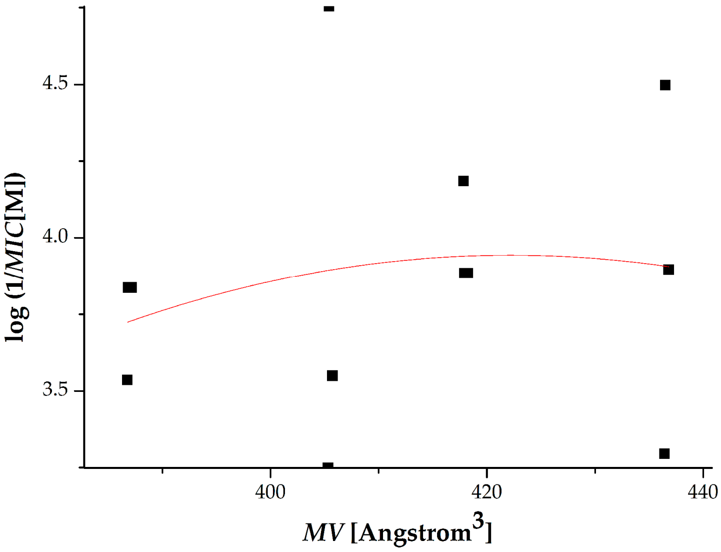

	Intercept	Intercept	B1	B1	B2	B2	Statistics
	Value	Standard Error	Value	Standard Error	Value	Standard Error	Adj. R-Square
B	-27.21877	82.3508	0.14762	0.40056	-1.74826E-4	4.86319E-4	-0.17631

Figure S4. The relationship between the activity (in the log (1/*MIC* [M]) units) of the tested compounds **5a–l** against *M. kansasii* DSM 44162 and the molecular volume (*MV*) data of particular bases **5aB–lB** investigated by a polynomial fitting analysis model.

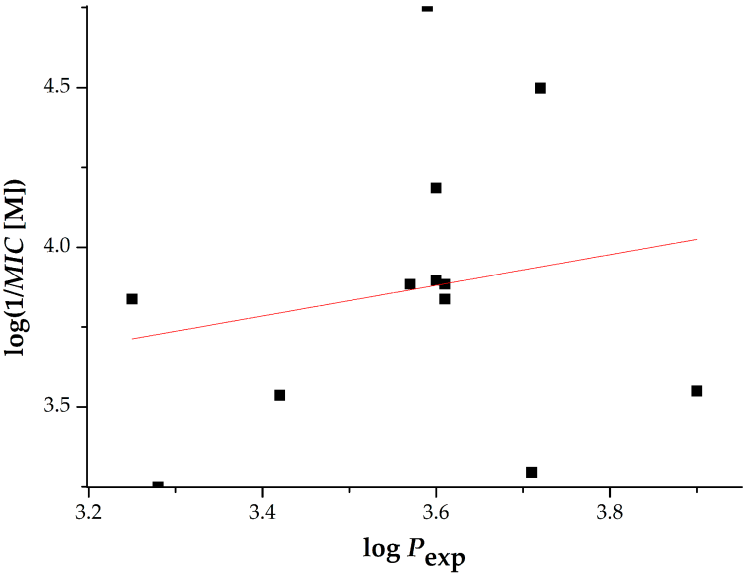

	Intercept	Intercept	Slope	Slope	Statistics
	Value	Standard Error	Value	Standard Error	Adj. R-Square
B	2.14745	2.72724	0.48145	0.76267	-0.05784

Figure S5. The relationship between the activity (in the log (1/*MIC* [M]) units) of the tested compounds **5a–l** against *M. kansasii* DSM 44162 and their lipophilicity (log P_{exp}) inspected by a linear fitting analysis model.

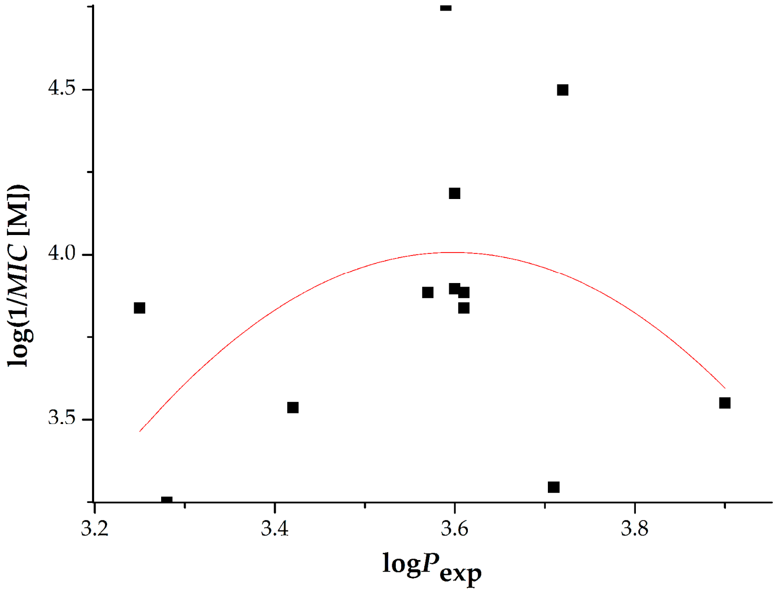

	Intercept Value	Intercept Standard Error	B1 Value	B1 Standard Error	B2 Value	B2 Standard Error	Statistics Adj. R-Square
B	-54.30374	40.41357	32.41684	22.82672	-4.50538	3.2187	0.03475

Figure S6. The relationship between the activity (in the log (1/*MIC* [M]) units) of the tested compounds **5a–l** against *M. kansasii* DSM 44162 and their lipophilicity (log P_{exp}) inspected by a polynomial fitting analysis model.

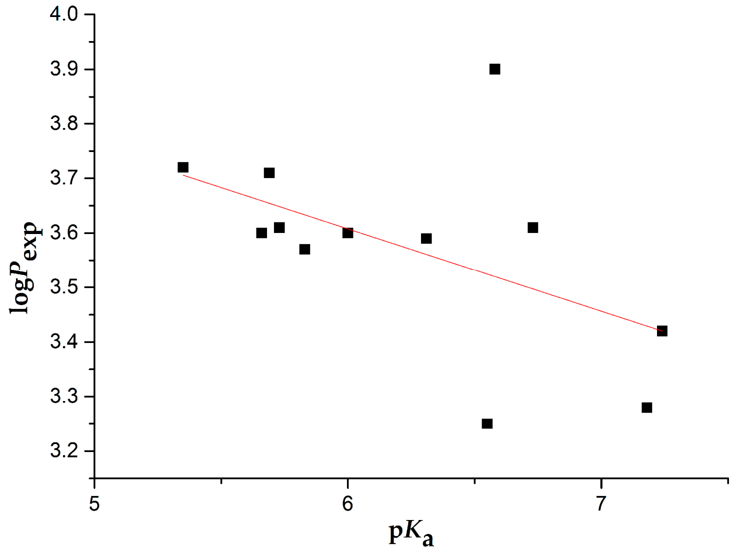

	Intercept	Intercept	Slope	Slope	Statistics
	Value	Standard Error	Value	Standard Error	Adj. R-Square
A	4.51534	0.49709	-0.15129	0.07933	0.19336

Figure S7. The relationship between experimentally observed pK_as and the log P_{exp} values of the analyzed compounds **5a–l**.

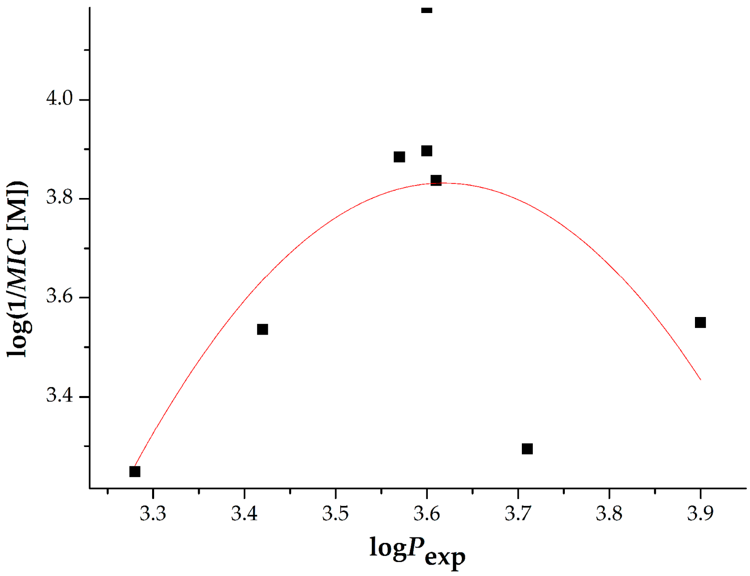

	Intercept	Intercept	B1	B1	B2	B2	Statistics
	Value	Standard Error	Value	Standard Error	Value	Standard Error	Adj. R-Square
B	-61.55586	32.63562	36.14509	18.21965	-4.99511	2.53969	0.23791

Figure S8. The relationship between the efficiency (in the log (1/*MIC* [M] units) of the tested 2- and 4-alkoxy substituted compounds (alkoxy = methoxy or ethoxy) against *M. kansasii* DSM 44162 and their lipophilicity (log P_{exp}) investigated by a polynomial fitting analysis model.

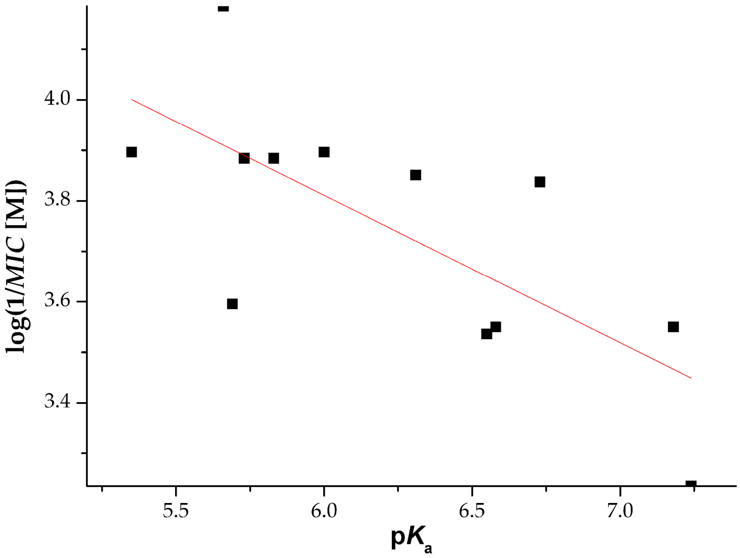

	Intercept	Intercept	Slope	Slope	Statistics
	Value	Standard Error	Value	Standard Error	Adj. R-Square
B	5.5636	0.55708	-0.2921	0.08891	0.47101

Figure S9. The relationship between the efficiency (in the log (1/*MIC* [M] units) of tested compounds **5a–l** against *M. marinum* CAMP 5644 and their electronic properties (pK_a) investigated by a linear fitting analysis model.

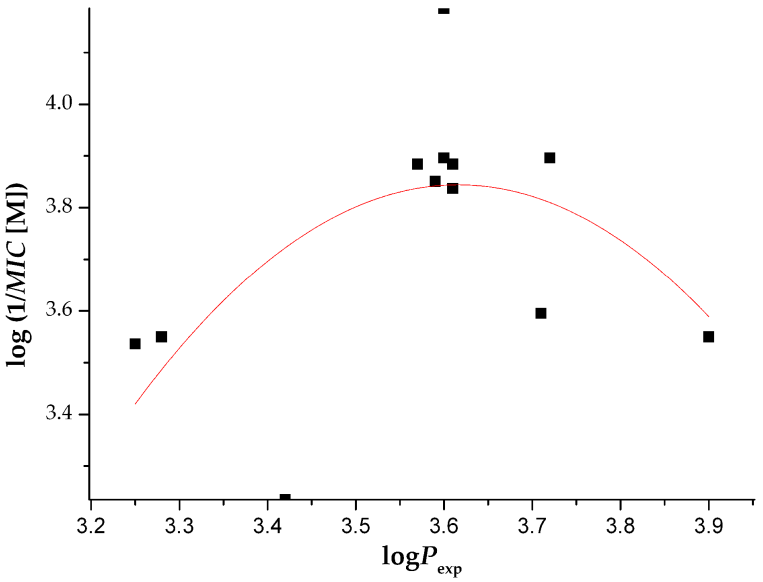

	Intercept	Intercept	B1	B1	B2	B2	Statistics
	Value	Standard Error	Value	Standard Error	Value	Standard Error	Adj. R-Square
B	-37.5678	20.20455	22.90471	11.4121	-3.16711	1.60917	0.23805

Figure S10. The relationship between the efficiency (in the log (1/*MIC* [M] units) of the tested compounds **5a**–**l** and their lipophilicity (log P_{exp}) investigated by a polynomial fitting analysis model.

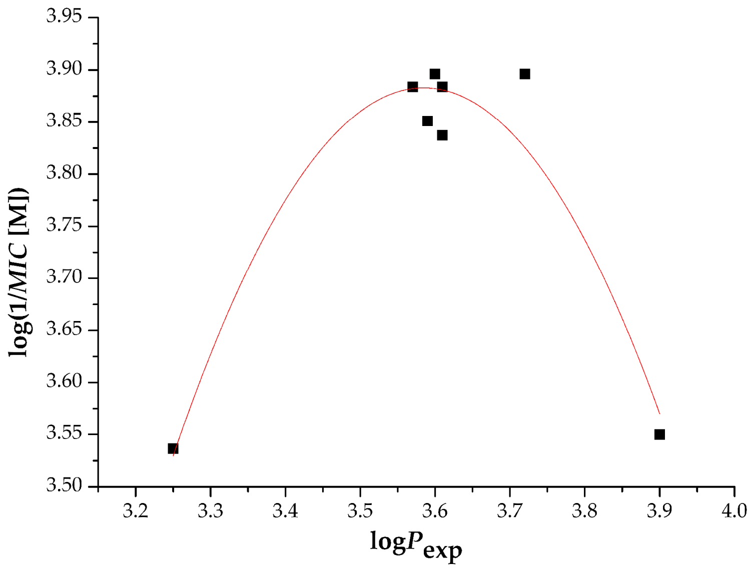

	Intercept	Intercept	B1	B1	B2	B2	Statistics
	Value	Standard Error	Value	Standard Error	Value	Standard Error	Adj. R-Square
B	-36.61505	4.20825	22.5939	2.36098	-3.15129	0.33084	0.92831

Figure S11. The relationship between the efficiency (in the log (1/*MIC* [M] units) of the tested 2- and 3-alkoxy substituted compounds (alkoxy = methoxy or ethoxy) against *M. marinum* CAMP 5644 and their lipophilicity (log P_{exp}) investigated by a polynomial fitting analysis model.

Table S1. The relationships between the independent variables (pK_a, *MV* and log P_{exp}) and the dependent variable log (1/*MIC* [M]) inspected for the *Mycobacterium kansasii* DSM 44162 strain.

Equation No.	Equation	Statistical Parameters					
		r	R^2	*F*	*RSS*	*Prob > F*	*n*
**(1)**	log (1/*MIC* [M]) = 5.970 (±1.262) − 0.337 (±0.201) × pK_a	0.375	0.141	2.80	1.73	0.125	12
**(2)**	log (1/*MIC* [M]) = −1.795 (±16.039) + 2.140 (±5.103) × pK_a − 0.196 (±0.403) × (pK_a)^2	0.265	0.070	1.41	1.68	1.413	12
**(3)**	log (1/*MIC* [M]) = 2.363 (±3.053) − 0.004 (±0.007) × *MV*	–	–0.074	0.24	2.16	0.633	12
**(4)**	log (1/*MIC* [M]) = −27.219 (±82.351) + 0.148 (±0.401) × *MV* − 1.7 × 10^{-4} (±1.7 × 10^{-4}) × (*MV*)^2	–	–0.176	0.18	2.13	0.842	12
**(5)**	log (1/*MIC* [M]) = 2.147 (±2.727) + 0.481 (±0.763) × log P_{exp}	–	–0.058	0.40	2.13	0.542	12
**(6)**	log (1/*MIC* [M]) = −54.304 (±40.414) + 32.417 (±22.827) × log P_{exp} − 4.505 (±3.219) × (log P_{exp})^2	0.187	0.035	1.20	1.75	0.346	12

Table S2. The relationships between the independent variables (pK_a, *MV* and log P_{exp}) and the dependent variable log (1/*MIC* [M]) inspected for the *Mycobacterium marinum* CAMP 5644 strain.

Equation No.	Equation	Statistical Parameters					
		r	R^2	*F*	*RSS*	*Prob > F*	*n*
**(7)**	log (1/*MIC* [M]) = 5.564 (±0.557) − 0.292 (±0.089) × pK_a	0.686	0.471	10.79	0.34	0.008	12
**(8)**	log (1/*MIC* [M]) = −0.613(±6.871) + 1.678 (±2.186) × pK_a – 0.156 (±0.173) × (pK_a)^2	0.679	0.461	5.70	0.31	0.025	12
**(9)**	log (1/*MIC* [M]) = 1.050 (±1.515) + 0.007 (±0.004) × *MV*	0.405	0.164	3.16	0.53	0.106	12
**(10)**	log (1/*MIC* [M]) = −43.894 (±38.346) + 0.225 (±0.187) × *MV* − 2.6×10^{-4} (±2.3×10^{-4}) × (*MV*)^2	0.440	0.194	2.33	0.46	0.153	12
**(11)**	log (1/*MIC* [M]) = 2.115 (±1.478) + 0.455 (±0.0.413) × log P_{exp}	0.138	0.019	1.21	0.62	0.296	12
**(12)**	log (1/*MIC* [M]) = −37.568 (±20.205) + 22.905 (±11.412) × log P_{exp} − 3.167 (±1.609) × (log P_{exp})^2	0.488	0.238	2.72	0.44	0.119	12

Table S3. Antimycobacterial screening *in vitro* (*MIC* expressed in the μg/mL units) of the compounds **5a–l** compared to the isoniazid (INH) and rifampicin (RIF) standards.

Compound	*MIC* (μg/mL)			
	MAP [1]	*MS* [2]	*MK* [3]	*MM* [4]
**5a**	250	256	64	64
**5b**	1000	256	64	64
**5c**	500	256	64	64
**5d**	1000	**64**	**16**	64
**5e**	500	256	**32**	**32**
**5f**	500	256	256	128
**5g**	1000	256	64	64
**5h**	500	256	128	128
**5i**	500	256	64	128
**5j**	250	128	**8**	64
**5k**	1000	256	128	256
**5l**	1000	256	256	128
**INH**	250	16.05	4	64
**RIF**	60	0.125	0.5	0.25

[1] *MAP, Mycobacterium avium* subsp. *paratuberculosis* CIT03; [2] *MS, M. smegmatis* ATCC 700084; [3] *MK, M. kansasii* DSM 44162; [4] *MM, M. marinum* CAMP 5644.

Table S4. The efficiency *in vitro* (*MIC* expressed in μg/mL units) of the compounds **5a–l** and the reference drugs ciprofloxacin (CPX), 5-flucytosine (5-FC), ampicillin (AMP) and amphotericin B (Amph. B) against some Gram-positive and Gram-negative bacterial strains and yeasts.

Compound	*MIC* (μg/mL)						
	SA [1]	*MRSA* [2]	*EC* [3]	*EF* [4]	*CA* [5]	*CP* [6]	*CK* [7]
**5a**	>256	>256	>256	>256	>128	>128	>128
**5b**	>256	>256	>256	>256	>128	>128	>128
**5c**	>256	>256	>256	>256	>128	>128	>128
**5d**	>256	>256	>256	>256	>128	>128	>128
**5e**	>256	>256	>256	>256	>128	>128	>128
**5f**	>256	>256	>256	>256	>128	>128	>128
**5g**	>256	>256	>256	>256	>128	>128	>128
**5h**	>256	>256	>256	>256	>128	>128	>128
**5i**	>256	>256	>256	>256	>128	>128	>128
**5j**	>256	>256	>256	>256	>128	>128	>128
**5k**	>256	>256	>256	>256	>128	>128	>128
**5l**	>256	>256	>256	>256	>128	>128	>128
CPX	0.25	>16	>16	>16	*nd*	*nd*	*nd*
5-FC	*nd* [8]	*nd*	*nd*	*nd*	1	8	0.125
AMP	2	>16	>16	>16	*nd*	*nd*	*nd*
Amph. B	*nd*	*nd*	*nd*	*nd*	0.54	1.08	0.54

[1] *SA, Staphylococcus aureus* ATCC 29213; [2] *MRSA*, methicillin-resistant *Staphylococcus aureus* 63718; [3] *EC, Escherichia coli* ATCC 25922; [4] *EF, Enterococcus faecalis* ATCC 29212; [5] *CA, Candida albicans* CCM 8261; [6] *CP, Candida parapsilosis* CCM 8260; [7] *CK, Candida krusei* CCM 8271; [8] *nd*, not determined, the compound was not used as the standard in the experiment.

Preparation of the Compounds 5a–l

The compounds under the study **5a–l** were synthesized according to the Scheme S1 and S2, as published in the research articles [30,65], which were listed in the References section of main text.

Those molecules were prepared by the reaction of 2-/3-/4-alkoxyphenyl isocyanates **1a–f** (alkoxy = methoxy or ethoxy) with (±)-oxiran-2-ylmethanol **2** in an anhydrous toluene. Given nucleophilic addition provided colorless (±)-oxiran-2-ylmethyl-2-/3-/4-alkoxyphenylcarbamates **3a–f** (Scheme S1). The compounds **3a–f** came under a nucleophilic addition by the reaction with 1-(3-trifluoromethylphenyl)piperazine **4a** and 1-(4-fluorophenyl)piperazine **4b**, respectively, to give solid or oily substances, 3-[4-(3-trifluoromethyl-/4-fluorophenyl)piperazin-1-yl]-2-hydroxypropyl -(2-/3-/4-alkoxyphenyl)carbamates **5aB–lB** (Scheme S2). The chemical structure of those intermediates was confirmed by the IR, ^{1}H-NMR and ^{13}C-NMR spectral analyses. Synthesized bases **5aB–lB** were converted into corresponding salts **5a–l**, 1-[3-(2-/3-/4-alkoxyphenylcarbamoyl)oxy-2 -hydroxy-propyl]-4-(3-trifluoromethyl-/4-fluorophenyl)piperazin-1-ium chlorides (Scheme S2). All those synthesized colorless target substances **5a–l** were fully characterized by the ^{1}H NMR, ^{13}C-NMR, IR and ESI-MS spectroscopy as well as by the CHN analyses. The observed results were in a full accordance with proposed structures [30,65].

The values of dissociation constant (pK_a; Table 1 in the main text) of final compounds **5a–l**, as a parameter, which described electronic and acidobasic features, and the partition coefficient readouts (log P_{exp}; Table 2), which expressed lipohydrophilic properties estimated in the octan-1-ol/phosphate buffer (pH = 7.4) system, were published in the research papers [24,25,30], listed in the References section of the main text.

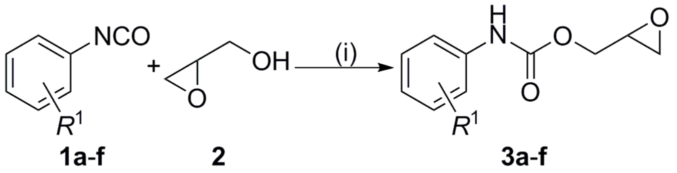

1a, **3a:** R^1 = 2-OCH_3, **1b, 3b:** R^1 = 2-OC_2H_5
1c, **3c:** R^1 = 3-OCH_3, **1d, 3d:** R^1 = 3-OC_2H_5
1e, **3e:** R^1 = 4-OCH_3, **1f, 3f:** R^1 = 4-OC_2H_5

Scheme S1. The synthesis of the reaction intermediates, (±)-oxiran-2-ylmethyl-2-/3-/4-alkoxyphenylcarbamates **3a–f**. *Reagents and conditions:* (**i**) dissolving of the intermediates in a dried toluene, the heating for 10 h at 65 °C.

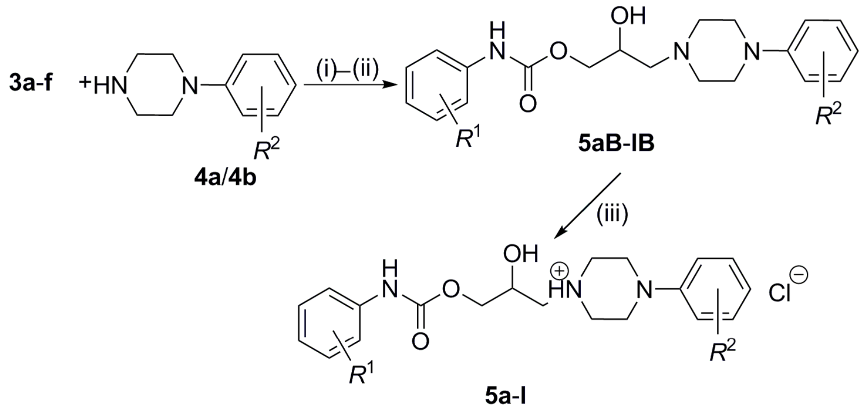

4a: R^2 = 3′-CF_3, **4b:** R^2 = 4′-F
5aB, **5a:** R^1 = 2-OCH_3, R^2 = 3′-CF_3, **5bB**, **5b:** R^1 = 2-OC_2H_5, R^2 = 3′-CF_3
5cB, **5c:** R^1 = 3-OCH_3, R^2 = 3′-CF_3, **5dB**, **5d:** R^1 = 3-OC_2H_5, R^2 = 3′-CF_3
5eB, **5e:** R^1 = 4-OCH_3, R^2 = 3′-CF_3, **5fB**, **5f:** R^1 = 4-OC_2H_5, R^2 = 3′-CF_3
5gB, **5g:** R^1 = 2-OCH_3, R^2 = 4′-F, **5hB**, **5h:** R^1 = 2-OC_2H_5, R^2 = 4′-F
5iB, **5i:** R^1 = 3-OCH_3, R^2 = 4′-F, **5jB**, **5j:** R^1 = 3-OC_2H_5, R^2 = 4′-F
5kB, **5k:** R^1 = 4-OCH_3, R^2 = 4′-F, **5lB**, **5l:** R^1 = 4-OC_2H_5, R^2 = 4′-F

Scheme S2. The synthetic route of the final compounds, 1-[3-(2-/3-/4-alkoxyphenylcarbamoyl)oxy-2-hydroxypropyl]-4-(3-trifluoromethyl-/4-fluorophenyl)piperazin-1-ium chlorides **5a–l**. *Reagents and conditions*: (**i**) the heating for 9 h, the stirring for 5 h at a room temperature; (**ii**) the solvents were removed *in vacuo*, crude intermediates were dissolved in chloroform, treated with water; organic fraction was collected, dried over an anhydrous magnesium sulfate and filtered; prepared bases were crystallized; (**iii**) an addition of saturated solution of hydrogen chloride in diethyl ether to individual solutions of the bases in chloroform, a continuous stirring for 2 h provided particular salts.
